# The parasitic nematode *Strongyloides ratti* exists predominantly as populations of long-lived asexual lineages

**DOI:** 10.1038/s41467-023-42250-1

**Published:** 2023-10-13

**Authors:** Rebecca Cole, Nancy Holroyd, Alan Tracey, Matt Berriman, Mark Viney

**Affiliations:** 1 School of Biological Sciences, University of Bristol, Bristol, BS8 1TQ UK; 2 Wellcome Sanger Institute, Wellcome Genome Campus, Hinxton, CB10 1SA UK; 3 Department of Evolution, Ecology and Behaviour, University of Liverpool, Liverpool, L69 7ZB UK; 4 Present Address: School of Infection & Immunity, University of Glasgow, 120 University Place, Glasgow, G12 8TA UK

**Keywords:** Population genetics, Ecological genetics, Parasite evolution

## Abstract

Nematodes are important parasites of people and animals, and in natural ecosystems they are a major ecological force. *Strongyloides ratti* is a common parasitic nematode of wild rats and we have investigated its population genetics using single-worm, whole-genome sequencing. We find that *S. ratti* populations in the UK consist of mixtures of mainly asexual lineages that are widely dispersed across a host population. These parasite lineages are likely very old and may have originated in Asia from where rats originated. Genes that underly the parasitic phase of the parasite’s life cycle are hyperdiverse compared with the rest of the genome, and this may allow the parasites to maximise their fitness in a diverse host population. These patterns of parasitic nematode population genetics have not been found before and may also apply to *Strongyloides* spp. that infect people, which will affect how we should approach their control.

## Abstract (web summary)

Nematodes are important parasites of people and animals. Here, the authors study the population structure of a nematode parasite of wild rats finding that it consists of mixtures of mainly asexual lineages widely dispersed across the host population.

## Introduction

Parasitic nematodes are important parasites of humans, livestock and other animals. In humans, parasitic nematodes are responsible for four of the twenty World Health Organization-defined Neglected Tropical Diseases^1^. In natural ecosystems, parasitic nematodes are highly abundant and a major force affecting host populations^2,3^, so understanding parasites’ biology is critical in understanding wider ecological patterns and processes. A study of the population genetics of parasitic nematodes can give important insights into their biology and patterns of transmission in host populations. This has been studied extensively in nematodes parasitizing livestock, for example, finding that they exist with very large effective population sizes, showing limited population genetic substructure, likely due to the high rate of livestock movement^4–6^. UK *S. ratti* populations appear to show a similar population structure^7^, and so in the present study our focus was on the high-resolution analysis of *S. ratti* populations. The population genetics of parasitic nematodes infecting humans has also been investigated to understand their host range and zoonotic potential^8,9^. In contrast, there has only been limited study of the population genetics and genomics of nematodes infecting natural, unmanaged species^10^.

*Strongyloides* spp. are a genus of parasitic nematodes with two species—*S. stercoralis* and *S. fuelleborni*—infecting some 100–600 million people worldwide^11,12^. *S. ratti* is a common parasite of rats, *Rattus norvegicus*^7^. In the *Strongyloides* spp. life cycle, hosts are infected by parasitic female worms only, which reproduce parthenogenetically^13^, producing eggs that pass out of the host in its faeces. Outside of the hosts, larvae can develop directly into infective larvae that then infect new hosts. Alternatively, and facultatively, larvae outside of the host can develop into a single generation of free-living adult males and females that reproduce sexually^14^, with their progeny then also developing into infective larvae to infect new hosts. Genetically, this means that *Strongyloides* reproduces by obligatory mitotic parthenogenesis inside the host and by facultative sexual reproduction outside of the host. The choice between asexual, direct development and sexual, indirect development is affected by environmental conditions (particularly the host immune response and the temperature outside of the host), but genotypes also differ in their propensity for these two developmental routes^15,16^. The extent of sexual *vs*. asexual reproduction in wild *Strongyloides* is not well known, though it appears to be rare in UK *S. ratti*^7^, perhaps more common in *S. ratti* from Japan^15^. This is in contrast to the development of the standard lab line of *S. ratti*^17^, though inadvertent selection in the laboratory may have selected for greater sexual development. In *S. stercoralis*, a parasite of people, development is predominantly asexual^18^.

*Strongyloides’* mode of reproduction is very likely to affect its population genetics. If reproduction is exclusively by mitotic parthenogenesis, then the only source of genetic variation is mutation, and such a population would consist of an assemblage of different genetic lineages. In this scenario, the mutation rate of a species is key in determining the extent of variation within a population. The occurrence of some sexual reproduction would allow genetic lineages of parasites to recombine, though the extent to which this will happen depends on the frequency with which sexual reproduction occurs. A three-locus study of *S. ratti* in the UK found that it consists of one interbreeding population, likely mainly reproducing by direct, asexual reproduction^7^. Populations of *Strongyloides* may change their balance of asexual and sexual reproduction during their history. A situation that we particularly consider here is that ancestral populations of *S. ratti* may have had more frequent sexual reproduction but then moved to principally asexual reproduction. Under this scenario, contemporary populations would have population genetic patterns consistent with asexual reproduction but also with some vestiges of ancestral sexual reproduction, such as some genotypes appearing to have a recombined ancestry.

*S. ratti* has a compact 43 Mbp genome consisting of 2 autosomes and an X chromosome, and its genome assembly is among the most contiguous for any nematode. This facilitates the population genomic analysis of wild *S. ratti*. Further, genomic, transcriptomic and proteomic analyses have identified genes that are putatively critical to *Strongyloides’* parasitic lifestyle. These were characterised in two ways: genes whose expression was significantly greater in the parasitic female stage compared with the free-living female stage; and genomic clusters of genes belonging to one of three families (encoding astacin-like metallopeptidases, CAP domain-containing proteins, acetylcholinesterases), which are gene families that have expanded as *Strongyloides* evolved to become parasites^19^.

In many, but not all, host-parasite systems, parasites can locally adapt to their host population, which enhances the fitness of those parasite genotypes^20^. The genes and gene products underlying parasite local adaption are not well known. In *Strongyloides*, the genes that have been shown to be central to its parasitism are at the interface between the parasite and its host and therefore may play a role in such adaptation. In such a scenario, these genes could be under different selection pressures compared with the rest of the genome and so may have population genetic patterns that differ from the rest of the genome.

Here we report the whole-genome, fine-scale population genomics of *S. ratti* in a wild rat population, describing how parasite genotypes are distributed among individual hosts. We find that these *S. ratti* populations exist as a mixture of asexual lineages and that these lineages are likely very long-lived. Comparison of these lineages with historical, geographically dispersed samples shows that some of these lineages may be very widely spatially dispersed. We find that genes and gene clusters critical to the parasitic phase of the *S. ratti* life cycle are genetically hyperdiverse compared with the rest of the genome. This hyperdiversity may contribute to *S. ratti*’s local adaption to its hosts within the context of it existing as long-lived asexual lineages.

## Results

### *S. ratti* is a common parasite of rats

**Fig. 1:**
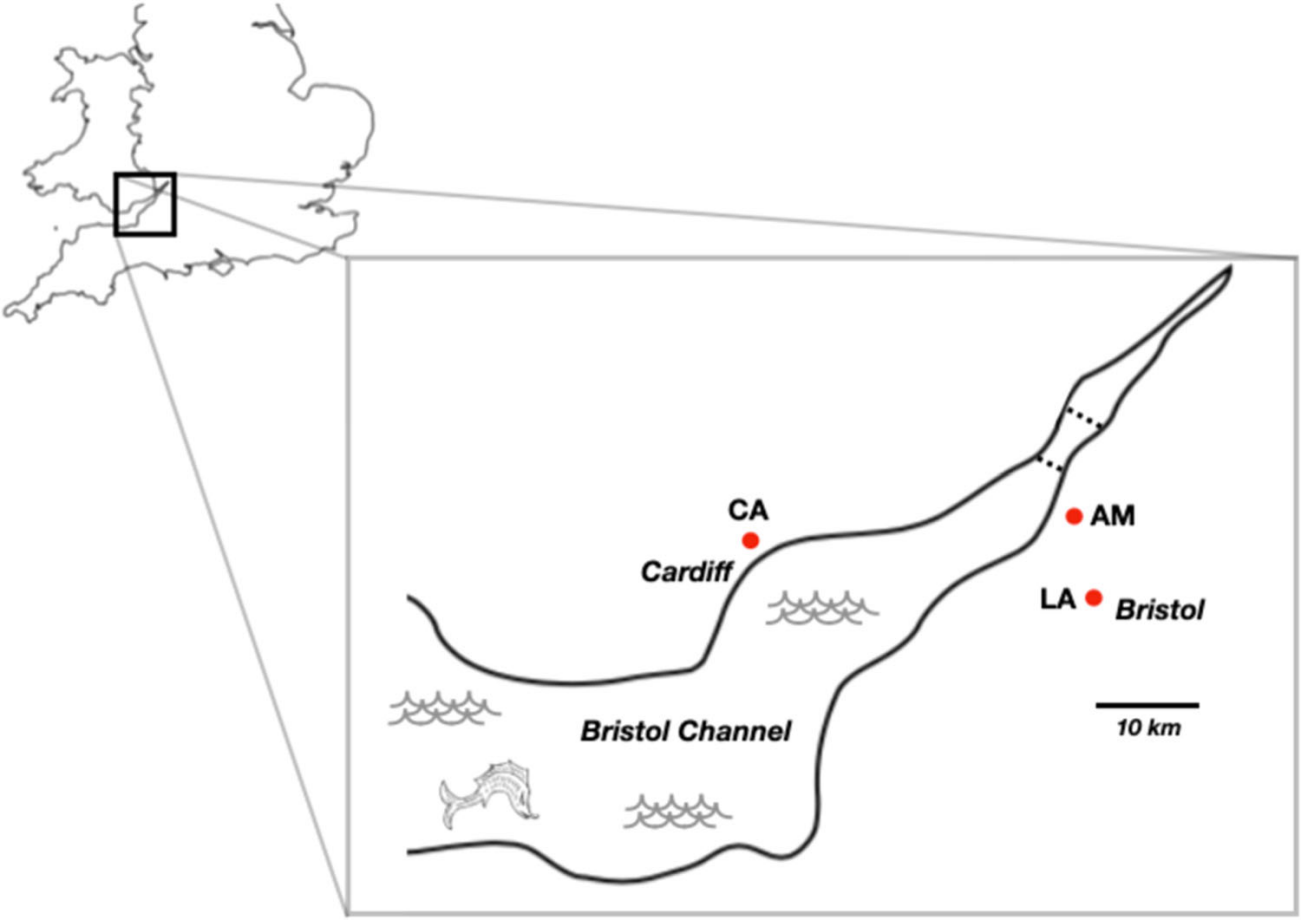
Map of sampling sites. Showing site LA near Bristol, AM on the English coast of the Bristol Channel, and CA on the Welsh coast of the Bristol Channel. Road bridges crossing the channel are shown as dotted lines; there is a train tunnel that is almost coincident with the southern bridge.

We sampled rat faecal pellets from three sites in the southwest UK (Fig. 1), from which we isolated 10,471 *S. ratti* infective larvae from 114 pellets (from a sample of 308). The proportion of infected faecal pellets significantly differed among the three sites (13, 47 and 62% at sites CA, AM and LA, respectively; χ^2^ = 48.9, *df* = 2, *P* < 0.0001) (Fig. 1; Supplementary Table 1), but is consistent with a previous report of a high prevalence of *S. ratti* in wild rats in the UK^7^. The number of *S. ratti* larvae per pellet ranged from 1 to 1730 (Supplementary Fig. 1). While culturing these faeces for *S. ratti*, we did not observe any sexual free-living adults. We genotyped rat faecal pellets to assign these to individual rats finding that 132 genotyped pellets belonged to 112 rats.

### *S. ratti* is partially genetically clustered at sample sites

We investigated the population genetics of *S*. *ratti* by analysing the whole-genome sequence of 90 individual infective larvae collected from the three sites. We identified 170,666 SNPs, giving an average density of 4.1 SNPs per kb. A total of 614 SNPs were tri-allelic, the remainder bi-allelic, with a ratio of 1.77 of transitions to transversions. Overall, 42% of SNPs were not in Hardy-Weinberg Equilibrium (HWE); for SNPs not in HWE, 77% had an excess of heterozygotes. Most SNPs (84%) had negative F_IS_ (inbreeding coefficient) values, consistent with heterozygote excess (Supplementary Fig. 2). This high level of heterozygosity is consistent with the absence of (or rare) sexual reproduction and recombination.

We analysed the pattern of parasite population genetic variation (1) within and among rat hosts and (2) within and among sampling sites, finding evidence of some differentiation of the parasites among the sample sites. The pairwise relatedness (Φ) among the 90 parasites was non-normal (Shapiro-Wilkes test for normalcy W = 0.902, *P* < 0.0001), suggesting genetic clustering among the worms at some level (Supplementary Fig. 3). *S. ratti* parasitic females reproduce by mitotic parthenogenesis^13^ and so siblings will be genetically identical save for individual-specific mutations. We did not detect putative sibling parasites within individual rats, despite sampling up to 4 parasites from each rat. (Supplementary Fig. 4). This suggests that faecal pellets commonly contained larvae of more than 4 genotypes. In faecal pellets containing ≥10 larvae, if there indeed had only been 4 genotypes present, then our chance of detecting the 4 would have been ≤0.19. It is more likely that faecal pellets contained worms of more than 4 genotypes. Specifically, we had a ≥0.50 chance of detecting 4 unique genotypes when: ≥6 genotypes were actually present in pellets containing 12–18 larvae; ≥7 genotypes were present in pellets containing 19–31 larvae. The average relatedness among pairs of parasites from the same rat and among parasites from different rats (Φ = 0.22 and 0.214, respectively) was not significantly different (t = −0.32, *df* = 108.81, *P* = 0.75). Thus, we conclude that individual rats contain genetically diverse parasites (consistent with our *S. ratti* RFLP genotyping, see “Methods” section), meaning that the genetic clustering of parasites is not at the level of the rat.

**Fig. 2:**
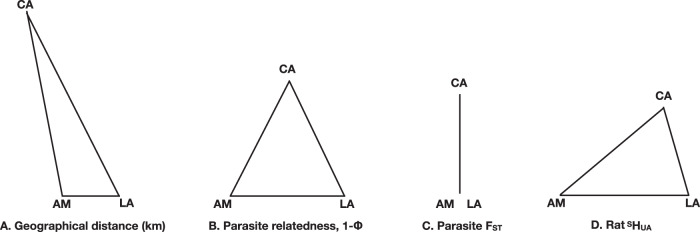
Physical and genetic differences among parasite and host populations. A schematic view of **A** Geographical distance among the sample sites, where direct distances between the sites are AM-LA = 9.2 km, CA-AM = 31.5 km, CA-LA = 33.5 km, **B** average pairwise relatedness (shown as 1-Φ) where Φ AM-LA = 0.244, CA-AM = 0.157, CA-LA = 0.162, **C** F_ST_ among parasites, from the three sample sites where, AM-LA = 0, CA-AM = 0.03, CA-LA = 0.03 and **D** ^S^H_UA_ among rats where, AM-LA = 0.21, CA-AM = 0.22, CA-LA = 0.15. Note that the scales of the metrics vary among the panels.

However, parasites from the same sample site were more closely related (mean same site Φ = 0.225; single, same site Φ, CA = 0.128, LA = 0.258, AM = 0.227) than parasites from different sample sites (Φ = 0.206) (t = −3.68, *df* = 3975.9, *P* ≤ 0.001) (Fig. 2). Overall, F_ST_ was very low (0.02), and indeed zero between parasites at the two English sample sites (Fig. 2). Together, these results show that there is some genetic clustering of *S. ratti* at the level of the sampling site, but not at the level of individual rats.

### *S. ratti* consists of divergent genetic clades that are widely distributed

**Fig. 3:**
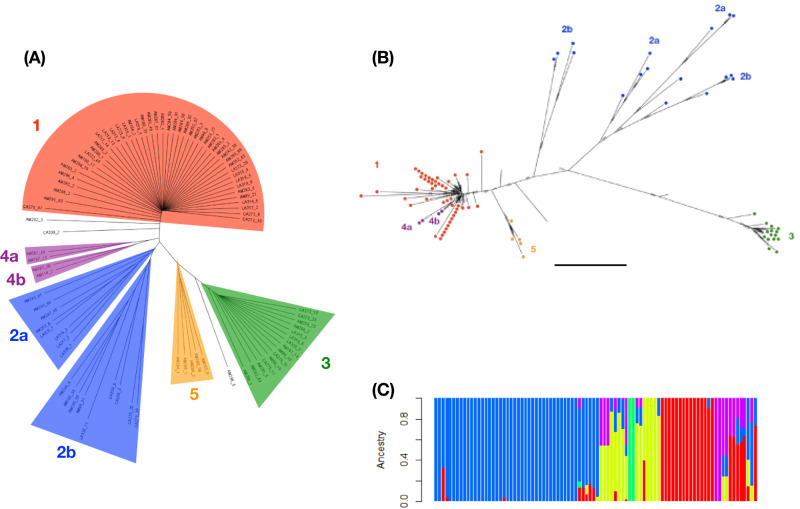
*S. ratti* consists of distinct genetic clades that are widely distributed. **A** A neighbour-joining dendrogram showing the five clades; **B** a maximum likelihood tree based on chromosome 1 where individuals are colour coded according to their clade membership in the neighbour-joining tree; the scale bar is 2 × 10^−^^4^ substitutions per site; chromosome-specific trees are shown in Supplementary Fig. 5; **C** the admixture of the 90 larvae for K = 5, which is the most strongly supported value of K; the order of individual worms and their neighbour-joining tree clade membership is shown in Supplementary Fig. 6. Note, the colour coding in (**C**) does not correspond to (**A**) or (**B**).

We examined the parasite diversity more closely by constructing neighbour-joining dendrograms. This suggested five parasite clades, with most worms (78 of the 90) in one of three clades (clades 1–3) (Fig. 3A). The structure of this tree, particularly the terminal divergence of clades (most obvious in clades 1 and 3) and the different clades is strongly suggestive of asexual reproduction by these parasites. Maximum likelihood trees also confirmed the existence of these clades (Fig. 3B; Supplementary Fig. 5). The precise relationship of parasites in the different clades is dependent on what part of the genome is used in the analysis, but this concurs with the whole-genome analysis of Fig. 3A. Importantly, the relationship between these parasites is consistent with mainly asexual reproduction. We examined the admixture among the 90 parasites, which most reliably grouped the parasites into five genetic groups, corresponding to the five clades defined by the neighbour-joining tree (Fig. 3C; Supplementary Fig. 6). Most worms (58 of 90, 64%) have no evidence of any admixture, consistent with an asexual origin. Of the remaining 32 parasites, most (18, 56%) were assigned to two clusters, which suggests recombination at some point in these genotypes’ history.

Analysis of linkage disequilibrium (LD) shows that there are large-scale blocks of LD and that it decays slowly over large genomic distances (of tens of thousands of bases), especially on the autosomes of clade 3 parasites (Supplementary Figs. 7–10). This is also consistent with absent or rare recombination in these populations. There is greater LD within Clade 1 and within Clade 3 (Supplementary Fig. 8) than in comparison of all parasites together (Supplementary Fig. 9), which is consistent with many generations of asexual reproduction from the common ancestors of Clade 1 and Clade 3, but with comparatively few generations of asexual reproduction preceding these ancestors (Supplementary Fig. 11).

Parasites belonging to the three main clades were present in all three sampling sites, in ratios expected based on the number of sequenced parasites from each sampling site (Fisher’s exact test, *P* = 0.14). Individual rats contained parasites from multiple clades; specifically, 11 rats contained parasites from two clades, and 3 rats contained parasites from three clades. Principal Component Analysis produced similar results, showing the clustering of parasites within clades and that these clades were dispersed across the three sampling sites (Supplementary Fig. 12).

Taken together, given that (1) no sexually reproductive stages were observed, (2) populations were more heterozygous than expected, (3) there is extensive LD, (4) there is terminal divergence of genotypes within clades and differences among genotypes of the clades (all of which are present in all three sampling sites) we conclude that these parasites are asexually reproducing and have been asexually reproducing for very many generations, but that rare, or historical, recombination occurs in these populations.

Analysis of Φ and F_ST_ within and among clades 1–3 is consistent with *S. ratti* being structured into sympatric, genetically distinct clades, with the three clades dispersed across the three sampling sites. Specifically, Φ was high within clades, especially clades 1 and 3 (0.43 and 0.45, respectively), but lower between clades; F_ST_ among the clades was 0.3 and similar (0.22–0.35) between clades (Table 1).

**Table 1:** F_ST_ and Φ among *S. ratti* clades 1, 2 and 3

	Clade 1	Clade 2	Clade 3
**Clade 1**	0.43	*0.22*	*0.35*
**Clade 2**	0.18	0.23	*0.23*
**Clade 3**	0.06	0.05	0.45

Pairwise F_ST_ is shown in italics above the diagonal, and Φ on and below the diagonal in non-italic text.

The population genetic structure of *S. ratti’s* mitochondrial genome consists of three main divergent genetic clades that are not strongly associated with sampling sites, which is consistent with the nuclear genome results. Specifically, in the mitochondrial genome, we identified 156 SNPs (average density 9.3 SNPs per kb), identifying 58 haplotypes among the 90 parasites. There was a strong, positive correlation between the pairwise similarities of mitochondrial and nuclear genomes (Mantel test, r = 0.76, *P* < 0.01) and between minimum spanning maps of mitochondrial haplotypes and neighbour-joining trees of nuclear genomes (Supplementary Fig. 13). The number of mitochondrial SNPs in same sampling site and different sampling site comparisons (23.6 and 24.2, respectively) were not significantly different (t = −0.98, *df* = 3771, *P* = 0.33).

We wanted to investigate if the rat host population genetic structure enforced a population genetic structure on the parasites because of the partitioning of parasites among individual hosts. To do this, we investigated the population genetics of the rats by genotyping faecal pellets at nine microsatellite loci (Supplementary Table 2). These loci were generally not in Hardy-Weinberg equilibrium (HWE); specifically, 8, 3 and 5 of the 9 loci at sites CA, AM and LA, respectively, were not (Supplementary Table 3). Rat allele frequencies differed markedly among sampling sites, consistent with restricted rat gene flow among the sites. The pairwise relatedness among rats was higher within sites than among sites (average Ritland and Lynch relatedness values 0.06 and −0.06, respectively), and the distribution of these relatedness values at each site had a right-hand skew (Supplementary Fig. 14), showing more closely related pairs of individual rats within sites than expected by chance. We could assign rats to each sample site based on allele frequencies with 89% accuracy. Shannon’s mutual information index (^S^H_UA_), which does not assume HWE^21,22^, shows that there is moderate genetic differentiation among rats from the three sites (Fig. 2). Together, these results—differences in allele frequencies among sample sites, higher within-site than among-site relatedness, high accuracy in assignment to sample site, moderate values of ^S^H_UA_—show that there is genetic differentiation among rats at the three sampling sites. The evidence of moderate genetic differentiation among rats at sample sites is broadly consistent with the geographical separation of the three sites and with limited migration of rats among the sites (Fig. 1), as has been observed with urban rats^23^. Notably, the genetic structure of the *S. ratti* population does not mirror that of its rat hosts.

We sought to understand the age of the *S. ratti* clades. We did this using the ML trees of chromosomes 1 and 2 to calculate the number of substitutions per site that have occurred since the last common ancestor (LCA) with the assumption of complete asexual reproduction of (1) clade 3 and (2) clades 1 and 3. Assuming neutrality and the *C. elegans* mutation rate of 2.7 × 10^−^^9^ per site per generation^24^, we calculate that there were an average of 18,401 (range 17,037–20,195) and 322,011 (range 213,185–393,589) generations since the LCA of clade 3 and of clades 1 and 3, respectively. To convert these generations to years, we assumed, conservatively, that there are two *S. ratti* generations per year in the wild, noting that *S. ratti* parasitic females have a maximum lifespan of a year when infecting immunodeficient laboratory rats^25^. On this basis, the LCA of clade 3 existed approximately 9000 years ago (i.e., 7000 BCE) and the LCA of clades 1 and 3 approximately 161,000 years ago, which is the Middle Pleistocene (now renamed the Chibanian). Given the dates, it is a reasonable scenario that the LCA of these *S. ratti* clades evolved outside of the UK and arrived in the UK with their hosts. *R. norvegicus* is thought to have arrived in mainland Europe from Asia about 1800 years ago (*c*. 200 CE) and into the British Isles from *c*.1750 CE^26,27^, where the black rat (*R. rattus*) had likely been since *c*. 1100 CE. Clades 1 and 3 may well have emerged in Asia, where *R. norvegicus* originated, and the LCA of clade 3 perhaps in mainland Europe.

We also investigated the spatial and temporal extent of the *S. ratti* clades by examining whole-genome sequences of 10 *S. ratti* isofemale lines derived from rats sampled from the UK and Japan between 1989 and 2012 (Supplementary Table 4). A neighbour-joining dendrogram of these isofemale lines and the 90 wild parasites (sampled in 2017/18) showed that these isofemale lines are not genetically distinct from the 90 parasites, occurring in clades 1, 2 and 4 (Supplementary Fig. 15). This observation—particularly of the two Japanese isofemale lines—is consistent with an Asian ancestry of the 90 contemporary UK genotypes, and more generally with the idea that *S. ratti* genotypes exist as long-lived, asexually maintained lineages.

In summary, these results show that *S. ratti* populations consist of mixtures of different genetic clades consisting predominantly of asexually maintained lineages. The *S. ratti* life cycle is obligately mitotically parthenogenetic^13^, with facultative sexual reproduction^14^; the population genetic structure that we have observed suggests that sexual reproduction (and so recombination) occurs very rarely, if at all, in these populations. Given the history of rats and their parasites, we suggest that ancestral *S. ratti* populations may have reproduced sexually but that they have since become largely or wholly asexual. Therefore the parasite lineages we observe in the UK are likely very long-lived—over thousands and tens of thousands of years—and widely distributed across the global host population. We observe no obvious geographical localisation of the different parasite clades, which might be expected with geographically-based host local adaptation. Indeed, given the likely asexual reproduction of these populations, it would therefore appear that *S. ratti* cannot genetically adapt to its local host populations except through mutational processes.

### *S. ratti* genes involved in parasitism are highly diverse

We next investigated the diversity of genes that *S. ratti* uses in the parasitic phase of its life cycle. We focussed on two sets of genes: (1) “parasitism genes”, which are genes whose expression is at least one log_2_-fold greater in the parasitic female morph compared with the free-living female morph, and (2) “expansion clusters”, which are genomic regions containing four or more genes encoding members of either astacin-like metallopeptidase, CAP domain-containing protein, or acetylcholinesterase families, which previous analyses have identified as having expanded with the evolution of parasitism in *Strongyloides*^19^.

**Fig. 4:**
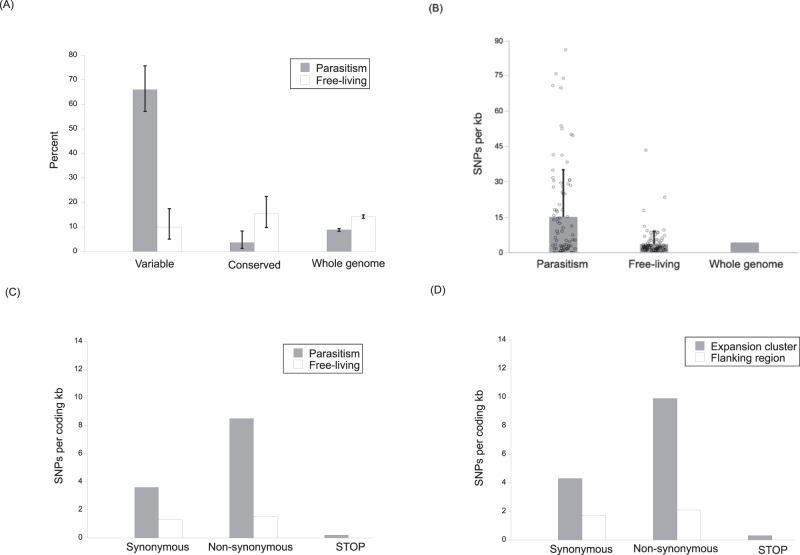
*S. ratti* genes involved in parasitism are highly diverse. **A** The percentage of genes in high diversity regions (≥200 SNPs per 10 kb), conserved regions (≤4 SNPs per 10 kb), or across the whole genome as a whole, that are parasitism or free-living genes. There were 107 genes in the variable regions, 137 in the conserved regions, and 12,464 across the whole genome. Parasitism and free-living genes are defined by Hunt et al.^19^. Errors bars are 95% binomial confidence intervals. **B** The average number of SNPs per kb (+1 SD) in parasitism genes (range 0–85.9), free-living genes (0–41.5), or across the genome as a whole. The SNP density for parasitism and free-living genes is calculated from coding sequence only; for the whole genome, all sequence data are used and so no SD is given. **C** The average density of SNPs of different effects in parasitism and free-living genes. **D** The average density of SNPs of different effects in expansion clusters and flanking regions.

Genetic diversity in *S. ratti* is not evenly distributed across the *S. ratti* genome (Supplementary Fig. 16), and we identified high SNP diversity regions consisting of ≥200 SNPs per 10 kb. We first asked how often parasitism or free-living (which are vice versa compared with parasitism genes) genes occurred in these high SNP diversity regions. We found that parasitism genes were significantly overrepresented within these high-diversity regions, compared with their representation in highly conserved genomic regions (≤4 SNPs per 10 kb region) or across the genome as a whole (Fig. 4A; Supplementary Data 1). In contrast, free-living genes were represented at the same rate in high-diversity regions, highly conserved regions, and across the genome as a whole (Fig. 4A).

Furthermore, two classes of genes—those encoding astacin-like metallopeptidases and CAP domain-containing proteins, both of which are associated with parasitism in *S. ratti*—were more common in these high-diversity regions, compared with the genome as a whole. Specifically, 5.6 and 11.8% (95% binomial confidence intervals 2.6–10.3% and 6.3–16.4%, respectively) of genes in these regions encode astacin-like metallopeptidase and CAP domain-containing proteins, which is higher than their representation across the whole genome (1.5 and 0.7%, respectively).

We compared the SNP density among the hundred most parasitic and most free-living genes, though we excluded six of the parasitism genes due to concerns about their underlying sequence assembly, leaving 94 parasitic genes (Supplementary Data 2 and 3). Parasitism gene SNP density was approximately four times that of free-living genes or that of the genome as a whole (Fig. 4B). The SNPs in these parasitism genes mainly code for non-synonymous substitutions rather than synonymous substitutions, which contrasts with free-living genes, where the rate of both types of SNPs occurred with similar frequency (Fig. 4C).

Concerning the expansion clusters, because of their repetitive nature and possible assembly and alignment errors, we manually curated these regions, excluding three whole clusters and other genes before our analysis (see “Methods”). Following this, we also found evidence of high genetic diversity in *S. ratti*’s expansion clusters, compared with the flanking regions (Supplementary Data 4). SNPs were three times more dense in the expansion clusters than in the flanking regions (SNP density (SD) per kb 15.9 (18.3) and 4.6 (7.2), in expansion clusters and flanking regions, respectively). Strikingly, SNPs within expansion clusters were approximately twice as likely to code non-synonymously rather than synonymously, unlike the flanking regions where these rates were similar (Fig. 4D); a pattern also seen for each individual expansion cluster, except clusters 1 and 8 (Supplementary Data 1).

We thought about possible reasons for the elevated genetic diversity in genes involved in *S. ratti*’s parasitism. Parasites can locally adapt to their hosts to maximise parasite fitness, and we hypothesised that the evolution of the expansion clusters, independent of the rest of the genome, could be a means by which host local adaptation occurs, where the expansion clusters evolve differently from the rest of the genome. To investigate this, we created neighbour-joining trees based on individual expansion clusters to see if they strongly diverged from the whole genome-based trees. However, in these expansion cluster-specific trees, the whole genome-defined clades 1 and 3 were still strongly evident (Supplementary Fig. 17). We next measured the selection in the expansion clusters and their flanking regions but could also find no consistent evidence for diversifying selection in the expansion clusters compared with their flanking regions (Supplementary Table 5). These results therefore do not support the idea that the expansion clusters are locally adapting to host genotypes.

Together, these observations—that highly variable genomic regions have an over-representation of parasitism genes and of astacin-like metallopeptidase and CAP domain-containing coding genes; that parasitism genes have SNP densities that are higher than those of free-living genes; that parasitism genes have a comparative excess of non-synonymous-coding SNPs; that expansion clusters have higher SNP densities and an excess of non-synonymous-coding SNPs—show that in *S. ratti* there is a concentration of genetic diversity within genes and genomic regions that very likely play a key role in the parasitic phase of its life cycle.

## Discussion

Parasitic nematodes are ubiquitous parasites of animals and are partitioned among individual hosts between which they must transmit, and so parasite and host biology can affect their population genetic structure^4,10^. Understanding the population genetics of parasitic nematodes can give an insight into their population biology, which is poorly known outside of species infecting humans and livestock^10^.

For the facultatively sexual parasite of rats *S. ratti*, we have discovered that in the UK its population consists of a mixture of genetically diverse clades, where those clades are widely dispersed across host populations (and possibly on a global scale), with very little evidence of structuring across three host populations. The population genetic data are consistent with these parasites existing as wholly, or very largely, asexually reproducing parasite populations. By estimating the age of the last common ancestor of these clades, we find that these parasite lineages are very long-lived, existing over some thousands to tens of thousands of years. This result does raise the question of whether these different lineages should be considered as different species or subspecies. Considering the age of these lineages with the history of the spread of the brown rat from Asia into mainland Europe and then to the British Isles, a plausible scenario is that the *S. ratti* lineages originated in Asia, where these populations perhaps had more frequent sexual reproduction, and that they then spread to the UK, and likely the world. It is notable that the neighbour-joining dendrograms show Japanese-derived *S. ratti* isofemale lines among the UK-derived genotypes. More extensive global sampling of *S. ratti* genetic diversity is now warranted.

This is a pattern of population genetic variation that has not, as far as we are aware, been observed in a parasitic nematode before. The life cycle of *S. ratti* contains an obligatory asexual parthenogenetic stage^13^, as well as a facultative sexual stage^14^. We observed no sexual stages during our work, suggesting that sexual reproduction is very rare, or even absent, in these UK populations, consistent with previous observations^7,15^. The population genetics of the populations that we studied is also consistent with the absence of sexual reproduction. Other lines of *S. ratti* (from Japan and a long-term lab-adapted line originally isolated from the United States) show some development of free-living males and females, suggesting that some sexual reproduction may occur in wild *S. ratti* populations beyond the UK^15^. Even low levels of sexual reproduction can be effective in the spread of alleles in a population, especially with strong selection^28^.

In contrast to the parasites’ population genetic patterns, the host rat populations did show some evidence of population genetic differentiation among the different sample sites, consistent with restricted movement of rats between the sites. Studies of the population genetics of rats in cities have also shown evidence of limited dispersal of rats and population genetic differentiation among rats in different city regions^29^.

Understanding a parasite’s population genetic structure can be important in understanding the host range of a parasite, which is of applied interest for parasites of humans. For example, population genetic analysis has been used to understand the possibly changing host range of Guinea worm (*Dracunculus medinensis*) in human and dog hosts during sustained control efforts in human populations^30^. There is considerable interest in understanding the host range of *S. stercoralis* that infects people; specifically, *Strongyloides* in dogs has been considered to be a source of human infection^31^. If the population genetic structure we have observed with *S. ratti*—a mixture of asexually maintained clones widely distributed across host populations—also pertains to *S. stercoralis*, then there is likely to be considerable complexity in understanding the population genetics and host range of *S. stercoralis* genotypes. Where a species exits as a collection of asexually maintained lineages, then each lineage could diverge genetically, which raises the possibility that *S. stercoralis* could exist as a mixture of lineages, each with different host ranges, for example, some able only to infect people, some only able to infect dogs and some intermediate and indeed there evidence (largely based on 18S and mitochondrial *cox1* loci) of this in *S. stercoralis* in Cambodia^31^. Current approaches to studying the host range of *S. stercoralis* have commonly only used single or a few loci, which cannot resolve more complex patterns of population genetic variation. Whole-genome analysis (following whole-genome amplification) of *S. stercoralis* from people in Japan and Myanmar found differences between parasites from these two sources^32^. If a population genetic structure similar to *S. ratti’s* also occurs in *Strongyloides* spp. infecting people and livestock, then there will be consequences for the evolution of anthelminthic resistance. In the absence of sexual reproduction, anthelmintic resistance could evolve separately in different *Strongyloides* lineages but would not be passed among them. The effect of this might be to slow the development of anthelminthic resistance in *Strongyloides* populations, compared with obligatory sexually reproducing nematodes, though with *Strongyloides* sustained anthelminthic use would, of course, select against anthelminthic sensitive genotypes.

Genomic analyses of *Strongyloides* have discovered genes and gene families that play a critical role in the parasitic phase of its life cycle. We have discovered that in wild *S. ratti*, both parasitism genes and genes in expansion clusters are highly diverse compared with other genes. Of particular note, many of these SNPs in the parasitism genes and genes in expansion clusters we observed are predicted to code for non-synonymous substitutions, meaning that this genetic diversity may cause functional differences in the gene products. The parasitism genes and genes in expansion clusters are dominated by large gene families. Large families can allow genetic diversity to accumulate among gene family members because any potential negative fitness consequence of a mutation in one gene of a family could be effectively ameliorated by others in that family. In this way, large gene families can allow the exploration of genetic space. The expansion clusters have a complex, repetitive structure, and the priority now is to use long-read sequencing approaches to fully resolve all clusters and so more clearly understand the elevated genetic diversity that they contain. More clearly resolving these regions will also improve the accuracy of measuring the genetic diversity in these regions. Our observation of comparatively high genetic diversity in these genes is particularly interesting in light of recent observations of genetic diversity in the free-living nematode *Caenorhabditis* spp. Specifically, analysis of worldwide populations of *C. elegans* finds that genetic variation is concentrated in a number of genomic regions (56 and 19 kb mean and median size, respectively), with evidence suggesting that diversity in these regions is maintained by balancing selection^33^. In the *C. tropicalis* genome, genetic variation is also distributed heterogeneously across its genome, for example, with some 140 high genetic diversity classified regions extending for more than 30 kb^34^. While there is a superficial similarity between the patterns of genomically concentrated genetic diversity in two *Caenorhabditis* species and in *S. ratti*, the mechanisms generating these patterns might be different. Notably, in *S. ratti*, we did not find any evidence of diversifying selection in the expansion cluster genes. What these two *Caenorhabditis* studies and the present study do demonstrate is that detailed, whole-genome analysis of wild individuals is uncovering hitherto unexpected patterns of genomic diversity that are likely to exist in other taxa too, and which need to be investigated.

Parasites have commonly been found to locally adapt to their host populations^20,35^. If such a phenomenon was occurring in *S. ratti*, then it may be manifest as geographical clustering of parasite genotypes per se or geographical clustering of parasitism gene genotypes and/or expansion cluster genotypes. However, the dispersion of *S. ratti* genotypes and of the genetic diversity in parasitism genes and expansion clusters that we have observed does not show any suggestive signatures of such local adaptation to hosts.

Alternatively, mindful that the products of the parasitism genes and genes in expansion clusters interface with the host, some variants of these genes may give a parasite a fitness advantage when infecting certain host genotypes, compared with parasites with other variants. Each individual parasite’s suite of these genes may therefore represent a combination of different variants that have been selected for as these parasite lineages have, over their history, infected a range of host genotypes. In this scenario, these parasites are not locally adapted to their host genotypes per se but rather have available a set of gene variants that are appropriate for a wide range of already-encountered host genotypes. This idea—the so-called grey pawn hypothesis—that the aggregate effect of a large number of diverse genes is selected so as to cover a large phenotypic space has previously been suggested in trying to understand the very large number of *Caenorhabditis* spp. chemoreceptor-coding gene families^36^; this idea has also been applied to hookworm parasites^37^. It is clear that further research is needed to understand the evolution of large gene families, as well as the full biological significance of high levels of genetic diversity in genes underlying *S. ratti’*s parasitism.

Our whole-genome, single-worm analysis of wild *S. ratti* is a non-destructive method of sampling parasite genetic diversity, and the hope must be that these approaches are now expanded to other parasitic nematodes. Such analyses will likely uncover different patterns of population genetic variation in other species, and understanding this will more fully illuminate the interactions between parasites and hosts and so underpin a better understanding of the rich ecology of parasites. If *Strongyloides* infecting people has a similar population genetic structure, then this may have consequences for understanding *Strongyloides’* host range and zoonotic potential, as well as for the evolution of anthelmintic resistance.

## Methods

### Parasite and rat sampling

We sampled at three sites in the southern UK—Avonmouth (AV), Cardiff (CA) and Long Ashton (LA) (Supplementary Table 6, Fig. 1), collecting fresh rat faecal pellets, which were cultured at 19°C and visually inspected for *S. ratti* infective third-stage larvae, which were washed twice in distilled water, once in 1% w/v SDS, and then twice more in distilled water, before being stored at −80°C. We genotyped rat faecal pellets at 9 dinucleotide repeat microsatellite loci (Supplementary Table 2) that had previously been used with wild rats^29,38–40^, preparing DNA using the QIAamp DNA Stool Mini Kit (Qiagen)^41^.

### *S. ratti* genotyping

Of the more than 10,000 *S. ratti* larvae that we isolated from wild rats, we had to select a subsample for whole-genome sequencing. We did this mindful that *S. ratti* parasitic females reproduce by mitotic parthenogenesis^13^, such that in pellets containing more than one larva, those larvae may be genetically identical siblings. Alternatively, a rat may be infected with multiple genotypes of *S. ratti*, in which case there will also be genetically different larvae in individual faecal pellets. We used RFLP genotyping to initially assess the genetic diversity among larvae within individual faecal pellets^41^. This showed that *S. ratti* infrapopulations are typically composed of multiple, genetically distinct parasitic adults and so we concluded that whole-genome sequencing of multiple infective larvae from the same faecal pellet was unlikely to result in extensive resequencing of genetically identical siblings, and as such, would be informative both for measuring the genetic diversity within sampling sites as a whole, and for assessing the extent of genetic variation within infrapopulations^41^.

For whole-genome sequencing, larvae were lysed and DNA prepared^41^. Samples were quantified with Biotium Accuclear Ultra high sensitivity dsDNA Quantitative kits using Mosquito LV liquid platform, Bravo WS and BMG FLUOstar Omega plate reader and cherrypicked to 200 ng/120 μL using a Tecan liquid handling platform. Cherrypicked plates were sheared to 450 bp using a Covaris LE220 instrument, and post-sheared samples were purified using Agencourt AMPure XP SPRI beads on Agilent Bravo. WSLibraries were constructed using the NEB Ultra II custom kit on an Agilent Bravo WS automation system. PCRs were set up using KapaHiFi Hot start mix and IDT 96 iPCR tag barcodes on an Agilent Bravo WS automation system and then purified using Agencourt AMPure XP SPRI beads on Beckman BioMek NX96 liquid handling platform. Libraries were quantified with Biotium Accuclear Ultra high sensitivity dsDNA Quantitative kit using Mosquito LV liquid handling platform, Bravo WS and BMG FLUOstar Omega plate reader. Libraries were pooled in equimolar amounts on a Beckman BioMek NX-8 liquid handling platform and libraries normalised to 2.8 nM ready for cluster generation on a c-BOT and loading on the Illumina X Ten platform. Sequencing reads from the libraries were aligned to the *S. ratti* reference assembly version 5_0_4^19^, taken from WormBase ParaSite release WBPS7 using Bowtie 2 version 2.2.9^42^ with default settings. We initially whole-genome-sequenced 225 *S. ratti* infective larvae at low depth of coverage and calculated the proportion of reads that aligned to the *S. ratti* genome, and used this metric to choose 90 libraries for further deep sequencing.

### Sequence analysis

We used BCFtools^43^ to identify SNPs using the criteria that they (1) fell on a nucleotide covered by at least 1000 reads (cumulative across all samples), (2) had a mean mapping quality of at least 20, and (3) had a QUAL score of at least 50. Among the 90 *S. ratti* genome sequences, nucleotides that were identical among all samples (but different from the ED321 reference genome) were removed. We sequenced to an average coverage of 96% of nucleotides (range 75.8–99.3%) and an average read depth of 68 (range 20–246; just 5 larvae had mean read depths of less than 30).

We noticed that the mean read depth on the X chromosome was 67.9% of the mean read depth on the two autosomes. We concluded that this was due to the GC content because (1) there was a significant correlation between read depth and GC content (Supplementary Fig. 18) and (2) that the X chromosome has a slightly lower GC content (19.7%) compared with the autosomes (22%)^19^.

Basic genetic diversity and population genetic statistics were calculated using VCFtools version 0.1.12^44^. Hardy-Weinberg equilibrium (HWE) was calculated considering only bi-allelic SNPs. Φ relatedness values^45^ of each pair of larvae were calculated using VCFtools and we used t-tests to compare Φ values. We also measured the differentiation among sites using the fixation index, F_ST_.

We generated neighbour-joining dendrograms using TASSEL 5.0^46^ and visualised these in FigTree Version 1.4.3. Clades within the neighbour-joining trees were identified by eye. Fisher’s exact tests, performed in R, were used to determine whether there were significant differences in the frequencies of these clades among sampling sites or sampling seasons.

We constructed maximum likelihood trees of the 90 parasites, producing consensus fasta sequences for each individual, but where an individual was heterozygous the reference allele was applied, with sequences aligned with MAFFT version 7^47^ using strategy FF-NST-1 for fast alignment, and maximum likelihood tree estimation performed using RaxML version 8.1.15^48^, using the general time reversible gamma model of substitution rate heterogeneity, and rapid bootstrapping with 100 replicates. We generated separate maximum likelihood trees for chromosome 1, the first 80 Mb of chromosome 2, the remainder of chromosome 2 and the two largest contigs of the X chromosome.

We conducted Principal Component Analysis using the R package pcadapt version 4.1.0^49^ using only loci with a minor allele frequency greater than 0.05. We investigated the admixture among the 90 parasite genotypes using ADMIXTURE version 1.3.0^50^. Due to computational constraints, for the 90 parasites, SNP data were first thinned so that no two SNPs were within 500 bp of each other, leaving a dataset of 35,559 SNPs. ADMIXTURE was run separately for k values 2-15.

We measured linkage disequilibrium (LD) among the 90 samples for the two autosomes and the two largest X chromosome scaffolds. We initially phased the genotype data into haplotypes using Beagle version 5.0^51,52^, where we used 100 burn-in iterations to generate an initial estimate of haplotype frequency, and a further 100 iterations were used to estimate the genotype phase for each SNP in each sample. Phasing is influenced by the effective population size (Ne), which is not known for *S. ratti*, but we estimated this as 50,000; otherwise, default Beagle parameters were used. We also undertook phasing using Shapeit version 2-r900^53^, where we used 100 burn-in iterations, 100 phasing iterations, and an estimated Ne of 50,000. For both, we used a window size of 0.5 Mb to estimate haplotypes. Only bi-allelic loci were used in Shapeit, but tri-allelic loci were also included in Beagle. We report the results from phasing using Beagle; Shapeit gave similar results.

To reduce computational time during linkage decay analysis, phased VCF files were thinned so that no two remaining loci were within 100 bp of one another. To perform linkage decay analysis, VCFTools was used to compare each SNP to each other SNP within a 50 kb window of it, with Pearson’s coefficient of correlation, r^2^, calculated for each pair. To measure LD across the whole genome, we further thinned the phased data so that no two SNPs were within 500 bp of each other when the analysis was repeated as above, except that this time each SNP was compared to every other SNP in the entire genome. We also repeated these analyses for subsets of parasites within the clades that we identified.

We also analysed the mitochondrial genomes (excluding one individual from site AM due to unexpectedly low mitochondrial read depth) and used Analysis of Molecular Variance (AMOVA), which was conducted in GenAlEx version 6.5^54,55^. Haplotype maps were generated in PopART version 1.7^56^ using the minimum spanning network method^57^, and maximum likelihood trees based on unique haplotypes were generated with RaxML version 8.1.15^48^, using the general time reversible gamma model of substitution rate heterogeneity and rapid bootstrapping with 100 replicates was applied. We calculated the proportion of SNPs shared among all pairs or worms and compared this to the nuclear Φ relatedness using a Mantel test.

We also used whole-genome sequence data of 10 isofemale lines derived from wild *S. ratti* (Supplementary Table 4), which we obtained from the European Variant Archive, study code PRJEB41 https://www.ebi.ac.uk/ena/data/view/PRJEB4163. Among the 90 wild *S. ratti* and 10 isofemale lines, there were 235,393 SNPs, of which 928 were tri-allelic, the remainder bi-allelic, with a ratio of 1.8 of transitions to transversions.

### Rat population genetic analysis

We only used data for faecal pellets that were successfully genotyped at 6 or more loci, resulting in 132 genotyped faecal pellets. Locus D12Rat42 was excluded from further population genetic analyses due to the low number of rats successfully genotyped at this locus. We used GenAlEx’s (version 6.5^54,55^) pairwise relatedness function to detect pellets with identical multilocus genotypes, which we took to have come from the same individual rat. We calculated Ritland and Lynch pairwise relatedness^58^, where each individual was compared with each other individual, and doubled these values to give a possible range of −1 to 1, from which we calculated the mean within-sample-site and mean among-sample-site relatedness. We determined the log-likelihood of the rat originating from each sampling site using GenAlEx to assign pellet genotypes to each sample site by comparing the multilocus genotype of each rat with the allele frequencies of each of the sampling sites (excluding the rat currently being investigated).

We used Shannon’s mutual information index (^S^H_UA_) to quantify the differences in allele frequencies among sampling sites and to estimate the number of effective migrants per generation. ^S^H_UA_ measures and is valid despite deviations from HWE within subpopulations^21,59^. ^S^H_UA_ ranges from 0 (indicating unhindered gene flow) to 1 (indicating a complete lack of gene flow).

We ensured (beyond visual identification) that none of the faecal pellets that we had genotyped were from species other than *R. norvegicus* by seeking to amplify the nine rat microsatellite loci from DNA isolated from other species that may potentially produce contaminating faecal material, specifically that from black rats (*R. rattus*), moles (*Talpa europaea*), and squirrels (*Sciurus carolinensis*). With one exception, none of these microsatellite loci successfully amplified from these species (the exception was locus D12Rat42 which did amplify *R. rattus* DNA), confirming that all successful genotypes were from *R. norvegicus*. As positive controls, we used primer pairs (1) Scv1 previously used to amplify *Sciurus* sp. DNA^60^, which successfully amplified our *Sciurus* sp. DNA, and weakly amplified *R. norvegicus* DNA, and (2) RodActin previously used to amplify *R. rattus* DNA^61^, which successfully amplified our *R. rattus* and *R. norvegicus* DNA.

### Parasitism and free-living genes and expansion clusters

We followed previous work that identified “parasitism genes”^19^. We excluded parasitism genes if they were part of an expansion cluster (below) and the underlying genome assembly was poor. We calculated 95% binomial confidence intervals for percentages from www.sample-size.net.

We define an “expansion cluster” as a genomic region containing four or more genes coding for members of one of three protein families (astacin-like metallopeptidases, CAP domain-containing proteins, or acetylcholinesterases) where there is not more than one other gene between any two genes of those families. This definition differs somewhat from and is more conservative than that used by ref. ^19^. As controls, we used “flanking regions”, which we define as the genomic region directly adjacent to the expansion cluster that is the same size as the cluster itself. Each expansion cluster has two flanking regions.

Lists of genes belonging to these three gene families were collated from ref. ^19^, and from this, we initially identified 15 expansion clusters. Because clusters 10 and 11 were very close to each other, cluster 10’s right flanking region was shortened to end where expansion cluster 11 began, and expansion cluster 11 was considered to not have a left flanking region. Similarly, to avoid overlap of cluster 11’s right flanking region and cluster 12’s left flanking region, we shortened cluster 12’s left flanking region to the start of cluster 11’s right flanking region. Across all expansion clusters there were 135 genes in total, of which 126 belonged to one of the three gene families: 46 encoding CAP domain-containing proteins, 70 encoding astacin-like metallopeptidases, and 10 encoding acetylcholinesterases, representing 51.7%, 38% and 33.3% of CAP domain-containing proteins, astacin-like metallopeptidase and acetylcholinesterase encoding genes, respectively, in the genome as a whole. Flanking regions collectively contained 216 protein-coding genes, the products of which had varying predicted functional descriptions.

Mindful that these expansion cluster regions were repetitive in nature, we checked their original reference genome assembly by realigning the sequencing reads originally used to build the reference assembly back to the reference, available at NCBI, BioProject code PRJEB2398, and then assessed the quality of these alignments using Gap5^62^. The repetitiveness of the sequence was examined via Dotplots with the software package Dotter^63^. Gene annotation schematics were retrieved from Ensembl’s ‘Region in Detail’ tool^64^, accessed via WormBase Parasite^65^ release 12 (https://parasite.wormbase.org/index.html) and added to the graphics produced by Gap5. Regions with poor mapping quality, unusually large distances between mate pairs and the occurrence of mate pairs facing opposite directions are suggestive of high rates of sequence misalignment. Peaks in read depth and fragment depth above background levels were evidence that multiple copies of a repetitive sequence were collapsed in the reference assemblies. Where expansion cluster genes or genes in flanking regions fell in poorly resolved reference assembly areas, these genes were excluded from further analysis.

Using this approach, we excluded expansion clusters 4, 11 and 13, and their flanking regions entirely and other genes within various clusters, resulting in 196 genes remaining, of which 61 were in expansion clusters and 135 were in flanking regions (Supplementary Data 4). Expansion clusters 6, 7, 8, 12 and 14 had no genes excluded. Three expansion clusters had genes that did not belong to one of the three target gene families and were excluded from analyses. Of the remaining 58 in the expansion cluster genes, 29 encoded CAP domain-containing proteins, 27 encoded astacin-like metallopeptidases, and 2 encoded acetylcholinesterases.

### Reporting summary

Further information on research design is available in the Nature Portfolio Reporting Summary linked to this article.

### Supplementary information

Supplementary Information

Reporting Summary

Supplementary Data 1

Supplementary Data 2

Supplementary Data 3

Supplementary Data 4

Supplementary Data 5

Description of Additional Supplementary Files

## Data Availability

The genome sequencing data for the 90 larvae used in this study are deposited in the European Nucleotide Archive (https://www.ebi.ac.uk/ena/browser/home), with accession numbers for individual larvae shown in Supplementary Data 5. The genomic variants (SNP data) for each sample have been submitted to the European Variation Archive as study PRJEB32744. Variants can be browsed in their genomic context at WormBase ParaSite (https://parasite.wormbase.org/Strongyloides_ratti_prjeb125/Tools/VEP).
