# Supplementary material for: The parasitic nematode Strongyloides ratti exists predominantly as populations of long-lived asexual lineages
Source: Nat Commun. 2023 Oct 13;14:6427. doi: 10.1038/s41467-023-42250-1 (PMC10575991; doi:10.1038/s41467-023-42250-1)

SUPPLEMENTARY INFORMATION

SUPPLEMENTARY TABLES

1. The occurrence of *S. ratti* in rat faecal pellets.
2. Rat microsatellite loci.
3. Population genetics of rat microsatellite loci.
4. Isofemale lines of *S. ratti* that were whole genome sequenced.
5. dN/dS ratios of expansion clusters and their flanking regions.
6. Sampling sites and times.

SUPPLEMENTARY FIGURES

1. Frequency distribution of the number of *S. ratti* infective larvae isolated from infected rat faecal pellets.
2. The distribution of F_{IS} values for all SNPs.
3. Histogram of Φ relatedness values among 90 *S. ratti* larvae.
4. The frequency distribution of the pairwise number of SNP differences among the 90 parasites.
5. Maximum likelihood trees of the 90 parasites.
6. ADMIXTURE analysis of the 90 parasites.
7. Linkage disequilibrium in the *S. ratti* genome.
8. Linkage disequilibrium in the *S. ratti* genome for clade 1 and 3 parasites.
9. Heatmaps of linkage disequilibrium in the *S. ratti* genome for clade 1 and 3 parasites.
10. Heatmaps of linkage disequilibrium in the *S. ratti* genome.
11. Model of clade ancestry
12. PCA analysis of *S. ratti* parasites.
13. Minimum spanning *S. ratti* mitochondrial haplotype maps.
14. Ritland and Lynch pairwise relatedness values of rats within sampling sites.
15. *S. ratti* neighbour-joining dendrograms of 10 isofemale lines and 90 larvae collected from the three sample sites.
16. The distribution of SNPs across the *S. ratti* genome.
17. Neighbour-joining dendrograms based on five expansion clusters.
18. Correlation of read depth and GC content for 90 *S. ratti* larvae.

SUPPLEMENTARY TABLES

Supplementary Table 1. The occurrence of *S. ratti* in rat faecal pellets. The proportion of infected pellets did not differ significantly among the seasons when the faecal pellets were collected (Chi-squared test, $\chi^2 = 6$, *df* = 3, P = 0.11).

Site and season	Number of pellets collected	Number (and %) of infected pellets	Number of larvae collected	Mean (SD) number of larvae per infected pellet
CA, Spring	35	7 (20%)	244	34.9 (24.3)
CA, Summer	32	5 (15.6%)	89	17.8 (22.1)
CA, Autumn	19	2 (10.5%)	256	128 (7.1)
CA, Winter	26	1 (3.8%)	6	6 (0)
**CA, All seasons**	**112**	**15 (13.4%)**	**595**	**39.7 (42.1)**
AM, Spring	11	8 (72.7%)	137	17.1 (23.5)
AM, Summer	75	23 (30.7%)	428	18.6 (29.3)
AM, Autumn	27	15 (55.6%)	3,044	202.9 (185)
AM, Winter	21	17 (81%)	5,067	298.1 (500.3)
**AM, All seasons**	**134**	**63 (47%)**	**8,676**	**137.7 (296.5)**
LA, Spring	27	14 (51.9%)	211	15.1 (23.1)
LA, Summer	11	7 (63.6%)	40	5.7 (3.7)
LA, Autumn	7	6 (85.7%)	360	60 (46.8)
LA, Winter	13	9 (69.2%)	589	65.4 (83.8)
**LA, All seasons**	**58**	**36 (62.1%)**	**1,200**	**33.3 (52.8)**
All sites, Spring	73	29 (39.7%)	592	20.4 (24.3)
All sites, Summer	118	35 (29.7%)	557	15.9 (25)
All sites, Autumn	53	23 (43.4%)	3,660	159.1 (162.4)
All sites, Winter	60	27 (45%)	5,662	209.7 (412.4)
**Total**	**304**	**114 (37.5%)**	**10,471**	**91.9 (226.9)**

Supplementary Table 2. Rat microsatellite loci. The forward primers are listed first and all primers are 5' to 3'; Length refers to the length in bp of the region amplified by the given primer sequences as determined from Rnor 6.0 (1.7.2014) release of the *R. norvegicus* genome; Fluorophore indicates the fluorophore used to label forward primers and thus PCR products.

**Locus**	**Primer sequences**	**Length**	**Fluorophore**	**Reference**
D3Rat159	CCAGGGATGAGTCCAAGGTA<br>CTGGTCTGCTTCCTCCAGTC	243	VIC	Steen *et al*., 1999
D4Rat59	GCAGTGTGTTTGGGGTAGCT<br>GCGGAATGATAGTTACTACGGC	180	FAM	Steen *et al*., 1999.
D6Cebr1	GGTTTGGTTGGGGAGAA<br>GTGCTGTCAGGGAAAGATGTA	223	NED	Giraudeau *et al*., 1999
D8Rat162	TCACTGGCAGCAATTTACCA<br>TCTGAGACCTCTTCAACTCTGTTG	249	VIC	Steen *et al*., 1999.
D10Rat105	ATCCAGCCAGAAAGCAAAAC<br>CTGGCTGAGTCCTGTCACAA	100	FAM	Steen *et al*., 1999.
D12Rat42	CAACCCAGTGTGTCAAACGT<br>GGGTTGGTGAAGCATTTTCA	128	VIC	Steen *et al*., 1999.
D14Rat110	AACATTGTCTTGCTTAGCCTCA<br>CTCCACCCACACACCACG	280	NED	Steen *et al*., 1999.
D18Rat11	GCCCAGGAGCTAAGTCTGATT<br>CCAGCCTCAGAGCCAATAAG	133	FAM	Steen *et al*., 1999.
D19Rat62	GTGCTAATGTGGGTGGCTTT<br>TGAATTCTACCATGCATCACAG	112	NED	Steen *et al*., 1999.

Supplementary Table 3. Population genetics of rat microsatellite loci. A. Genetic diversity of loci. Rats genotyped is the number of individual rats in which genotyping was successful; Allele number is the number of alleles identified at the locus; He and Ho are expected and observed heterozygosity, respectively; Site diversity is the proportion of allelic diversity that partitions within sites, as opposed to among sites, according to $^{S}H_{UA}$. Locus D12Rat42 was not used in analyses due to the low genotyping success rate. **B. HWE of loci.** Locus shows the loci; Site is the sampling site; Rats genotyped is the number of rats that were genotyped; Alleles is the number of alleles detected; X^2 is the Chi-squared statistic testing whether the observed genotype frequencies match HWE expectations with the degrees of freedom (df) in parentheses. Those still significant after Bonferroni correction are shown in bold.

A.

**Locus**	**Rats genotyped**	**Allele number**	**He**	**Ho**	**Site diversity (%)**
D3Rat159	103	14	0.779	0.756	18
D4Rat59	105	12	0.822	0.652	8
D6Cebr1	109	14	0.752	0.356	13
D8Rat162	73	15	0.835	0.600	16
D10Rat105	112	8	0.696	0.617	13
D12Rat42	34	9	0.762	0.267	NA
D14Rat110	83	16	0.824	0.529	18
D18Rat11	112	10	0.686	0.319	19
D19Rat62	96	12	0.678	0.462	13

B.

**Locus**	**Site**	**Rats genotyped**	**Alleles**	**X^2 (df)**	**Probability**
**D3Rat159**	CA	45	7	41.8 (21)	0.01
	LA	17	11	89.9 (55)	0.01
	**AM**	**41**	**10**	**98.4 (45)**	**0.001**
**D4Rat59**	**CA**	**46**	**9**	**74.6 (36)**	**0.001**
	LA	15	9	39.1 (36)	0.03
	AM	44	10	67.2 (45)	0.05
**D6Cebr1**	**CA**	**45**	**11**	**100.7 (55)**	**0.001**
	**LA**	**18**	**10**	**87.6 (45)**	**0.001**
	**AM**	**46**	**6**	**105.1 (15)**	**0.001**
**D8Rat162**	**CA**	**25**	**10**	**120.1 (45)**	**0.001**
	LA	13	8	28.3 (28)	0.45
	AM	35	10	67.7 (45)	0.05
**D10Rat105**	**CA**	**47**	**5**	**89.6 (10)**	**0.001**
	**LA**	**19**	**6**	**64 (15)**	**0.001**
	**AM**	**46**	**7**	**94.2 (21)**	**0.001**
**D12Rat42**	**CA**	**15**	**9**	**71.8 (36)**	**0.001**
	LA	5	4	12 (6)	0.06
	AM	14	6	33.1 (15)	0.01
**D14Rat110**	**CA**	**34**	**11**	**112.4 (55)**	**0.001**
	LA	16	9	45.2 (36)	0.14
	**AM**	**33**	**11**	**113.6 (55)**	**0.001**
**D18Rat11**	**CA**	**47**	**8**	**66.6 (28)**	**0.001**
	**LA**	**19**	**5**	**34.5 (10)**	**0.001**

	**AM**	**46**	**9**	**141 (36)**	**0.001**
**D19Rat62**	**CA**	**39**	**9**	**115.2 (36)**	**0.001**
	LA	17	7	40.8 (21)	0.01
	AM	40	5	17.9 (10)	0.06

Supplementary Table 4. Isofemale lines of *S. ratti* that were whole genome sequenced.

Isofemale line	Isolation year	Origin
ED36	1990	Hampshire, UK
ED43	1989	Edinburgh, UK
ED53	1990	Kagoshima, Japan
ED132	1990	Kagoshima, Japan
ED336	1995	Berkshire, UK
ED391	1989	Wiltshire, UK
ED399	1989	Sussex, UK
ED405	1989	Sussex, UK
ED428	2012	Bath, UK
ED438	2012	Bath, UK

Supplementary Table 5. dN/dS ratios of expansion clusters and their flanking regions. The dN/dS ratio for genes in expansion clusters and in parentheses the number of genes, N. We calculated dN/dS ratios by analysing parasites from clades 1 and 3 (**Figure 3**), treating each as separate populations.

Expansion cluster	Expansion cluster mean dN/dS ratio (N)	Flanking region mean dN/dS ratio (N)
**2**	0.257 (1)	2.088 (2)
**3**	0.454 (5)	0.735 (6)
**6**	1.373 (1)	0.408 (1)
**7**	3.419 (2)	0.278 (3)
**14**	0.322 (3)	1.242 (1)

Supplementary Table 6. Sampling sites and times.

Site (code)	Coordinates	Type
Cardiff (CA)	51°29'54"N 3°07'25"W	Industrial
Avonmouth (AM)	51°30'43"N 2°40'15"W	Industrial
Long Ashton (LA)	51°26'08"N 2°38'41"W	Farm
**Season**	**Sampling start date**	**Sampling End date**
Spring	2.2017	3.2017
Summer	6.2017	6.2017
Autumn	9.2017	11.2017
Winter	12.2017	2.2018

SUPPLEMENTARY FIGURES

Supplementary Figure 1. Frequency distribution of the number of *S. ratti* infective larvae isolated from infected rat faecal pellets. Uninfected pellets (N = 178) are not shown. The x-axis is in increments of 20 larvae, with only the upper limit shown, and only alternate increments labelled.

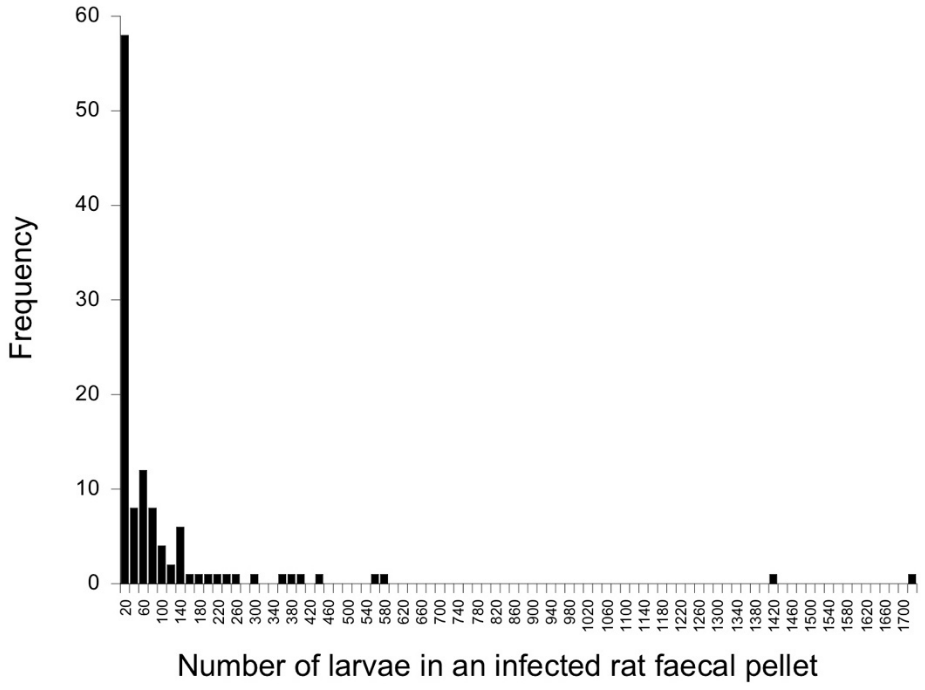

Supplementary Figure 2. The distribution of F_{IS} values for all SNPs. The x-axis is in 0.1 increments with only the lower (for negative) or higher (for positive) limits shown; category 0 is where F_{IS} = 0.

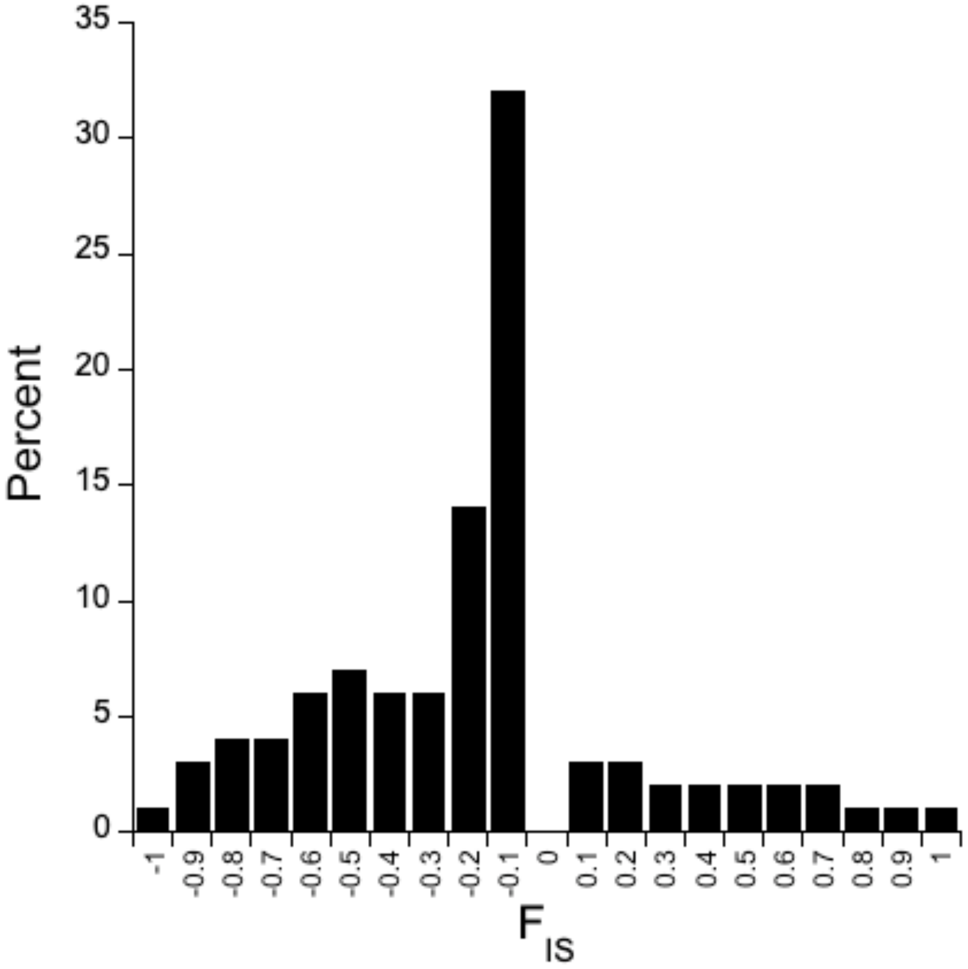

Supplementary Figure 3. Histogram of Φ relatedness values among 90 *S. ratti* larvae. The x-axis is in increments of 0.05 with only the upper limit shown.

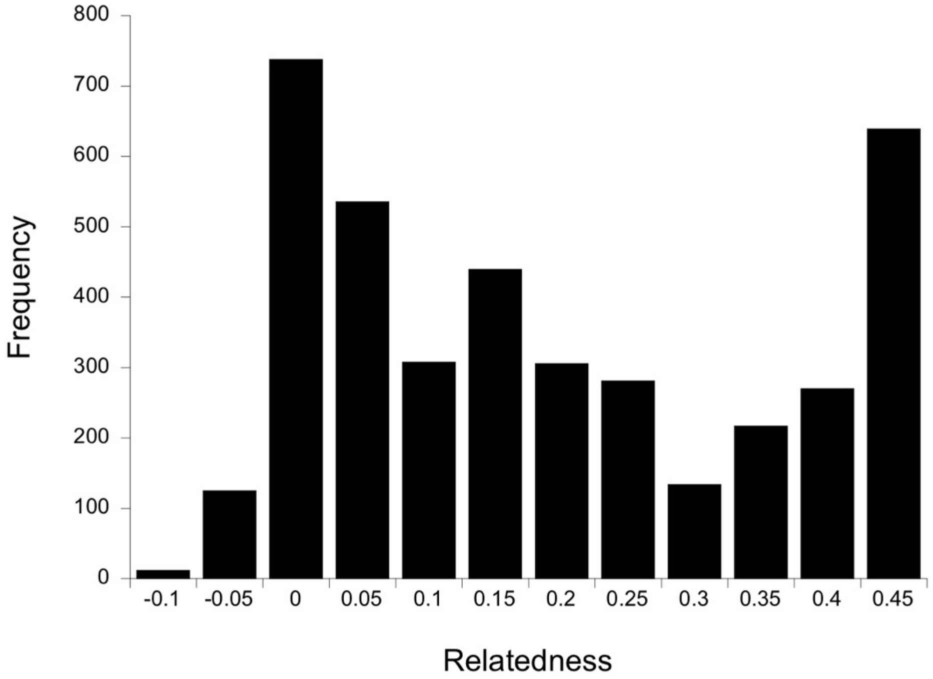

Supplementary Figure 4. The frequency distribution of the pairwise number of SNP differences among the 90 parasites. The x-axis is in categories of 10,000, save for the first three which are 0-1,000, 1,000-5,000, 5,000-10,000; in all the lower end of the range is shown. The frequency is 1 in the first category.

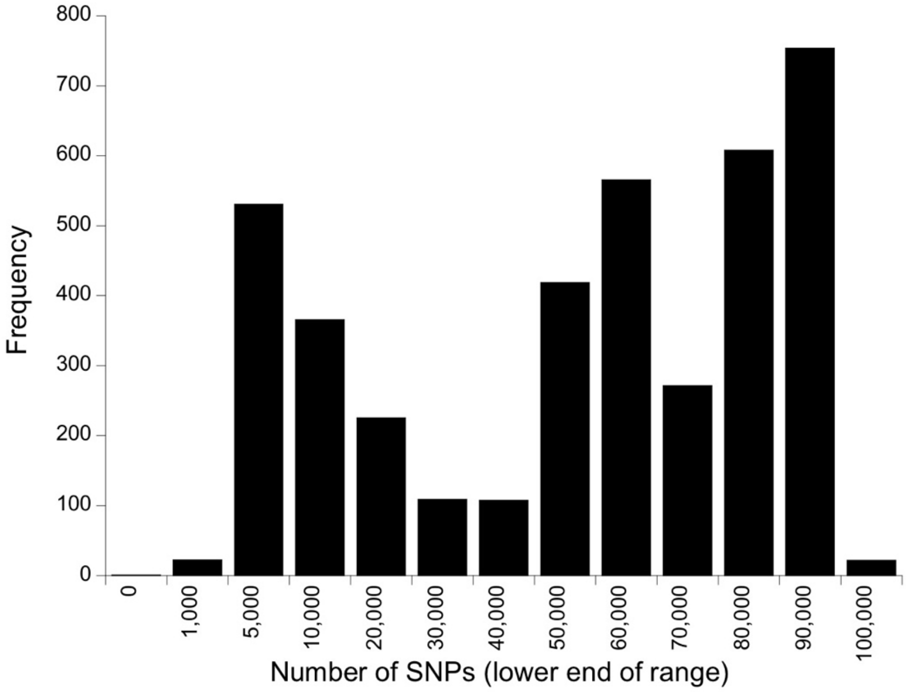

Supplementary Figure 5. Maximum likelihood trees of the 90 parasites. In each tree, the support for each node is shown, and the clade membership of the worm is shown by the coloured dots, which correspond to the neighbour-joining tree (**Figure 3**). Trees are calculated based on analysis of chromosome 1 only, part 1 of chromosome 2, part 2 of chromosome 2, part 1 of the X chromosome, and part 2 of the X chromosome. In all the scale bar is in units of substitutions per site.

Chromosome 1

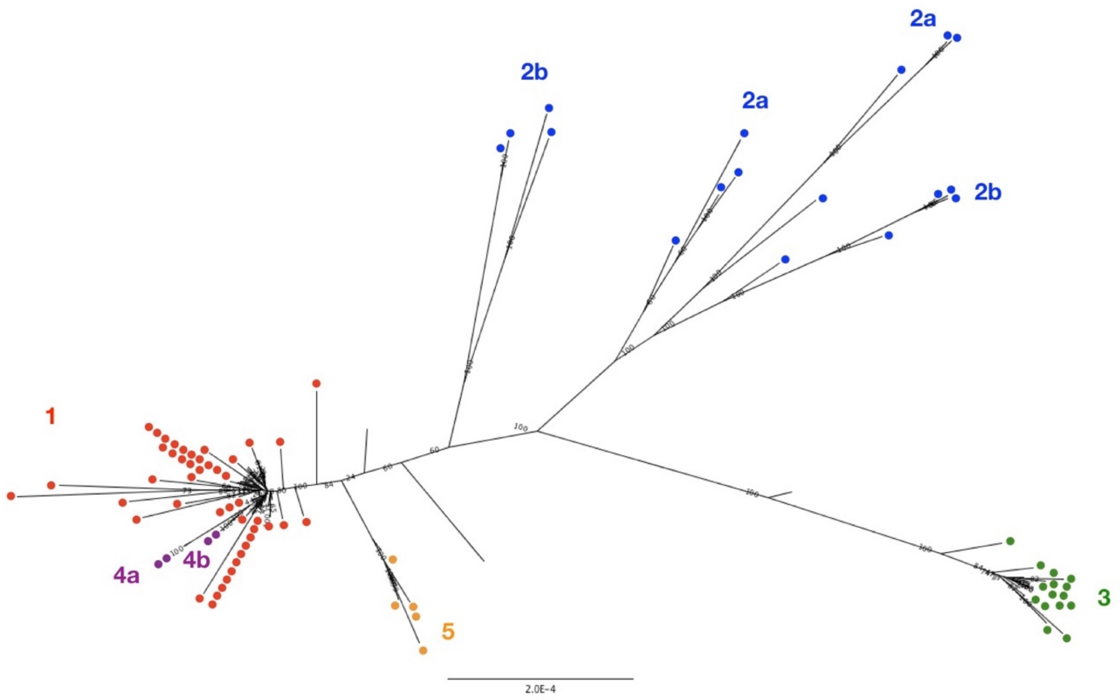

Chromosome 2, part 1

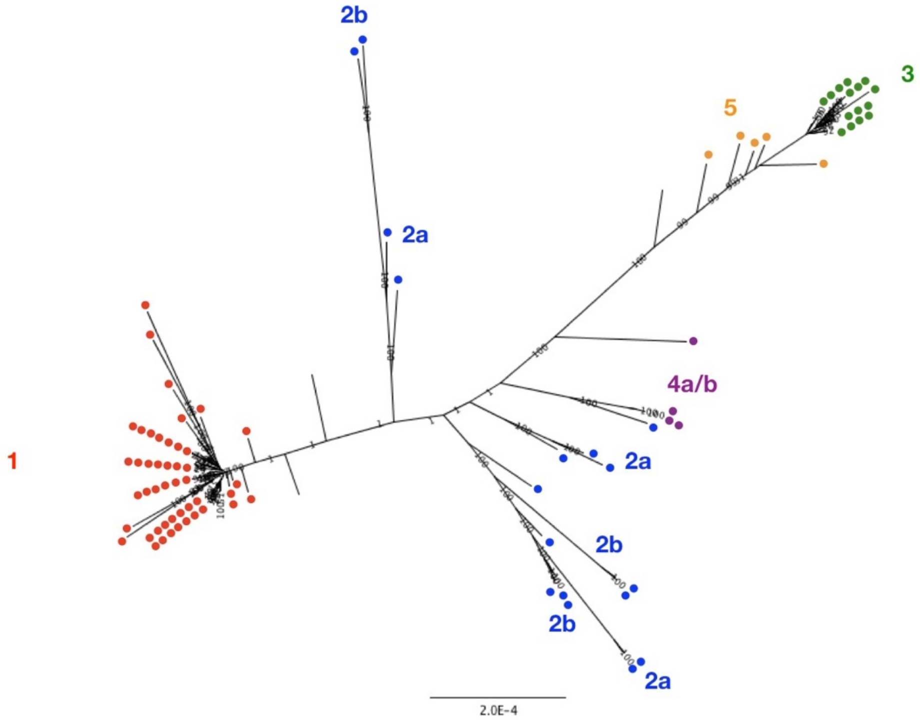

Chromosome 2, part 2

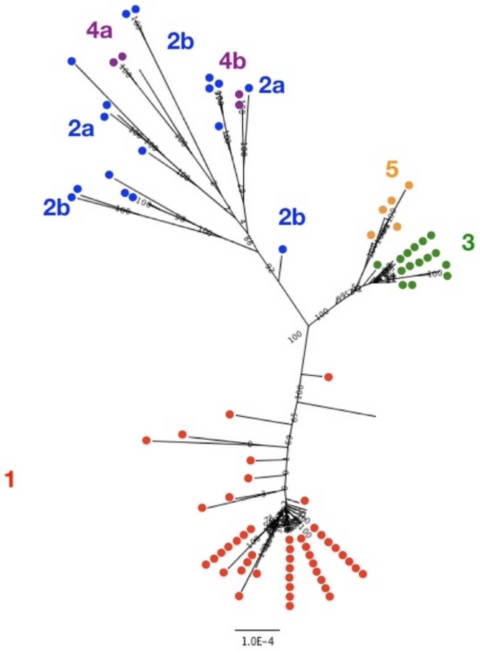

Chromosome X, part 1

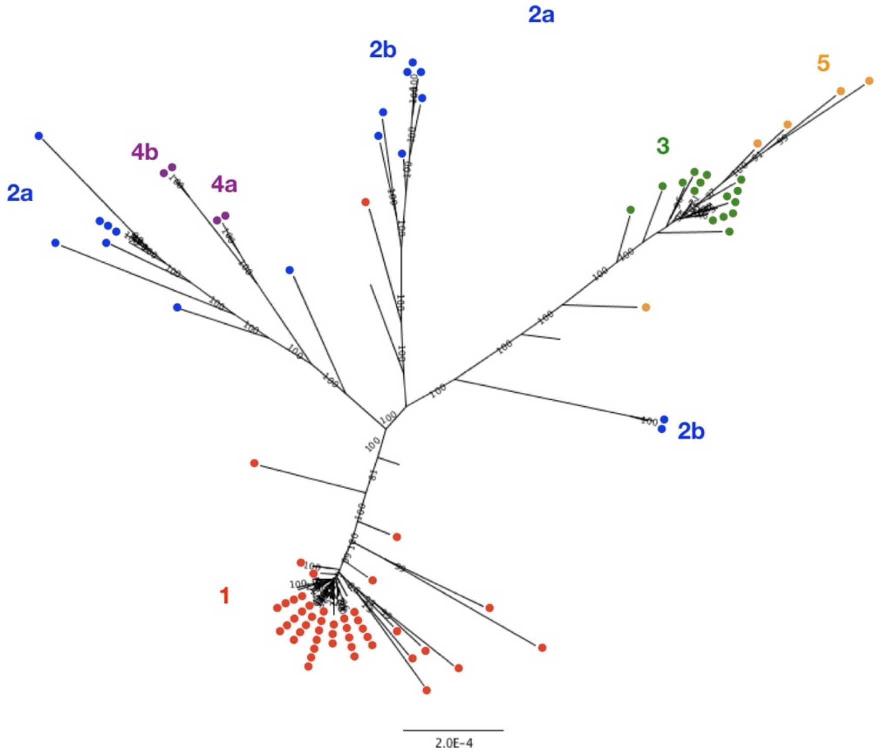

Chromosome X, part 2

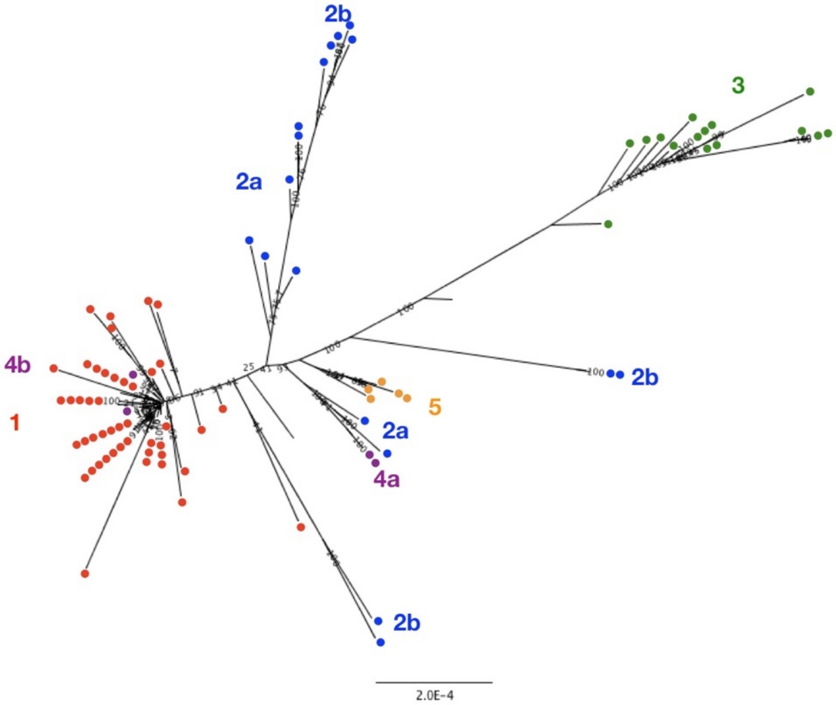

Supplementary Figure 6. ADMIXTURE analysis of the 90 parasites. The ADMIXTURE diagrams are shown for K = 2, 3, 4, 5 (with cross validation errors of 0.22965, 0.16255, 0.16335, 0.16136, respectively). For K = 6, only five groups are detected. All diagrams have the same right to left order of worms, which is shown in a Table at the end of the figure.

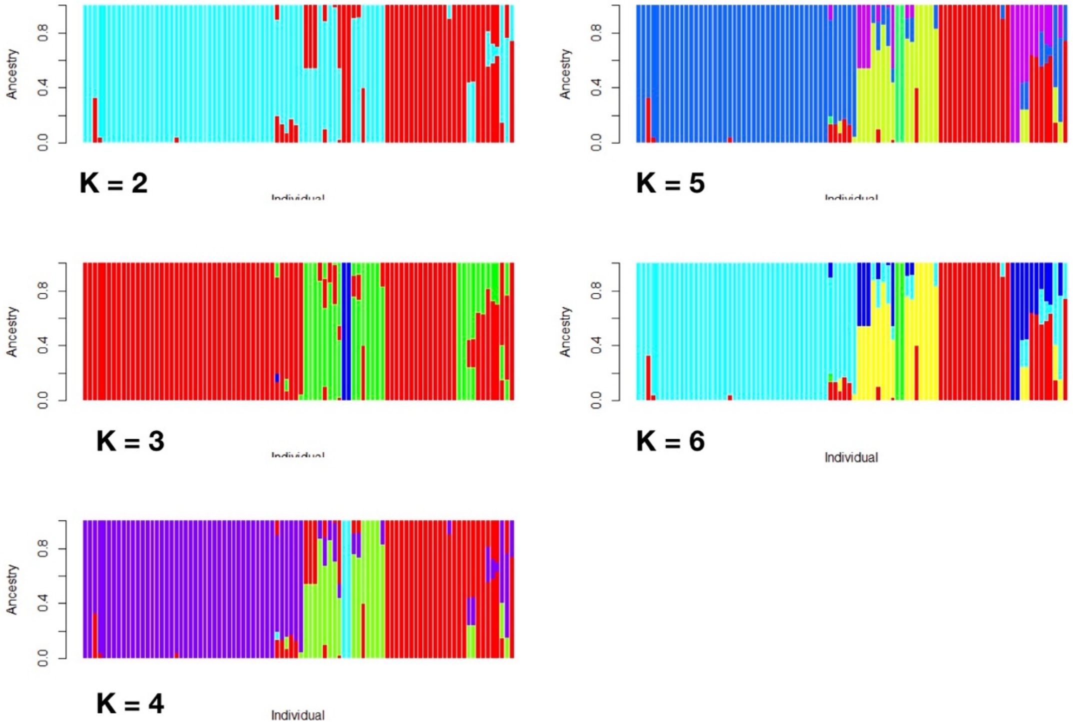

Position in ADMIXTURE diagram	Worm designation	NJ tree clade membership
Far left	CA273_8	1
	CA273_30	1
	CA275_47	1
	LA217_14	1
	LA219_13	1
	LA222_45	1
	LA223_8	1
	LA223_29	1
	LA14_5	1
	LA315_1	1
	LA315_4	1
	LA316_4	1
	LA316_5	1
	LA317_2	1
	LA319_5	1

	LA319_8	1
	AM99_8	1
	AM99_21	1
	AM100_37	1
	AM190_17	1
	AM194_1	1
	AM242_30	1
	AM252_62	1
	AM253_77	1
	AM280_49	1
	AM282_1	1
	AM282_67	1
	AM283_1	1
	AM283_2	1
	AM283_3	1
	AM283_4	1
	AM283_5	1
	AM284_59	1
	AM284_60	1
	AM284_61	1
	AM286_88	1
	AM286_92	1
	AM287_67	1
	AM288_1	1
	AM288_2	1
	AM291_93	1
	AM292_2	1
	AM293_2	1
	AM294_2	1
	AM296_4	1
	AM299_79	1
	CA338_1	2a
	LA217_5	2a
	LA319_3	2a
	LA320_1	2a
	AM245_61	2a
	AM253_6	2a
	AM287_65	2a
	AM293_94	2b
	CA273_38	2b
	CA275_20	2b
	CA338_4	2b
	CA338_6	2b
	LA320_11	2b

	AM99_31	2b
	AM100_35	2b
	AM100_50	2b
	AM294_4	2b
	CA273_10	3
	CA273_26	3
	CA275_11	3
	CA275_51	3
	LA315_3	3
	LA319_6	3
	LA320_5	3
	AM99_19	3
	AM99_30	3
	AM187_14	3
	AM258_25	3
	AM281_5	3
	AM282_63	3
	AM296_1	3
	AM298_2	3
	AM187_12	4a
	AM287_58	4a
	AM114_2	4a
	AM287_56	4a
	AM105_9	5
	AM280_56	5
	AM294_1	5
	AM295_1	5
	AM296_2	5
	AM292_3	Not in clade
	CA338_2	Not in clade
Far right	AM296_5	Not in clade

Supplementary Figure 7. Linkage disequilibrium in the *S. ratti* genome. LD shown as r^2 values. X1 and X2 are the largest and second largest X chromosome scaffolds, respectively.

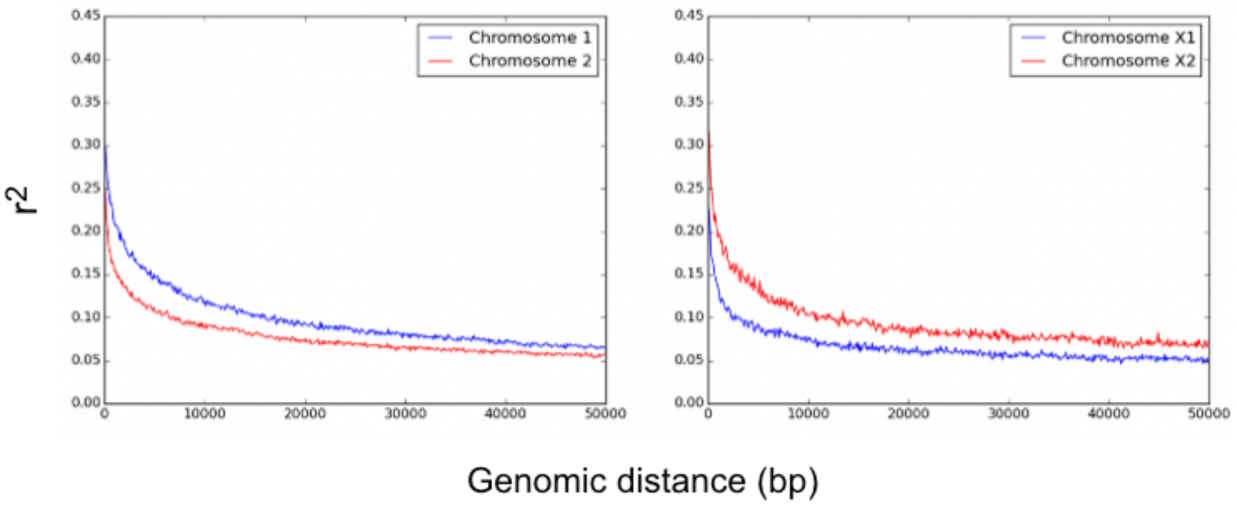

Supplementary Figure 8. Linkage disequilibrium in the *S. ratti* genome for clade 1 and 3 parasites. LD is shown as r^2 values for (A) clade 1 and (B) clade 3 parasites. X1 and X2 are the largest and second largest X chromosome scaffolds, respectively.

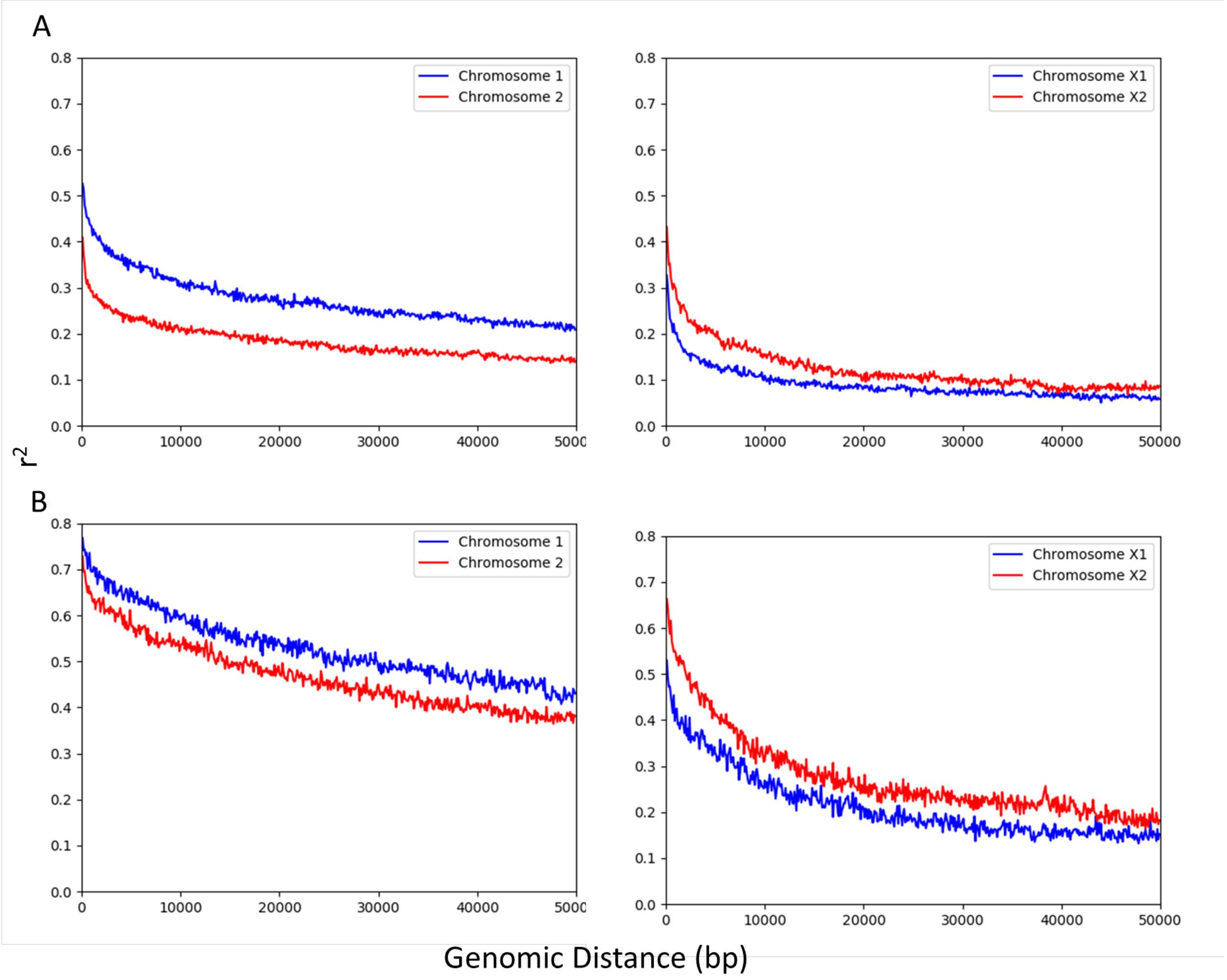

Supplementary Figure 9. Heatmaps of linkage disequilibrium in the *S. ratti* genome for clade 1 and 3 parasites. LD is shown as r^2 values on a coloured scale for (A) clade 1 and (B) clade 3. X1 and X2 refer to the largest and second largest scaffolds of the X chromosome, respectively. The inserts enlarge regions to show areas of high LD.

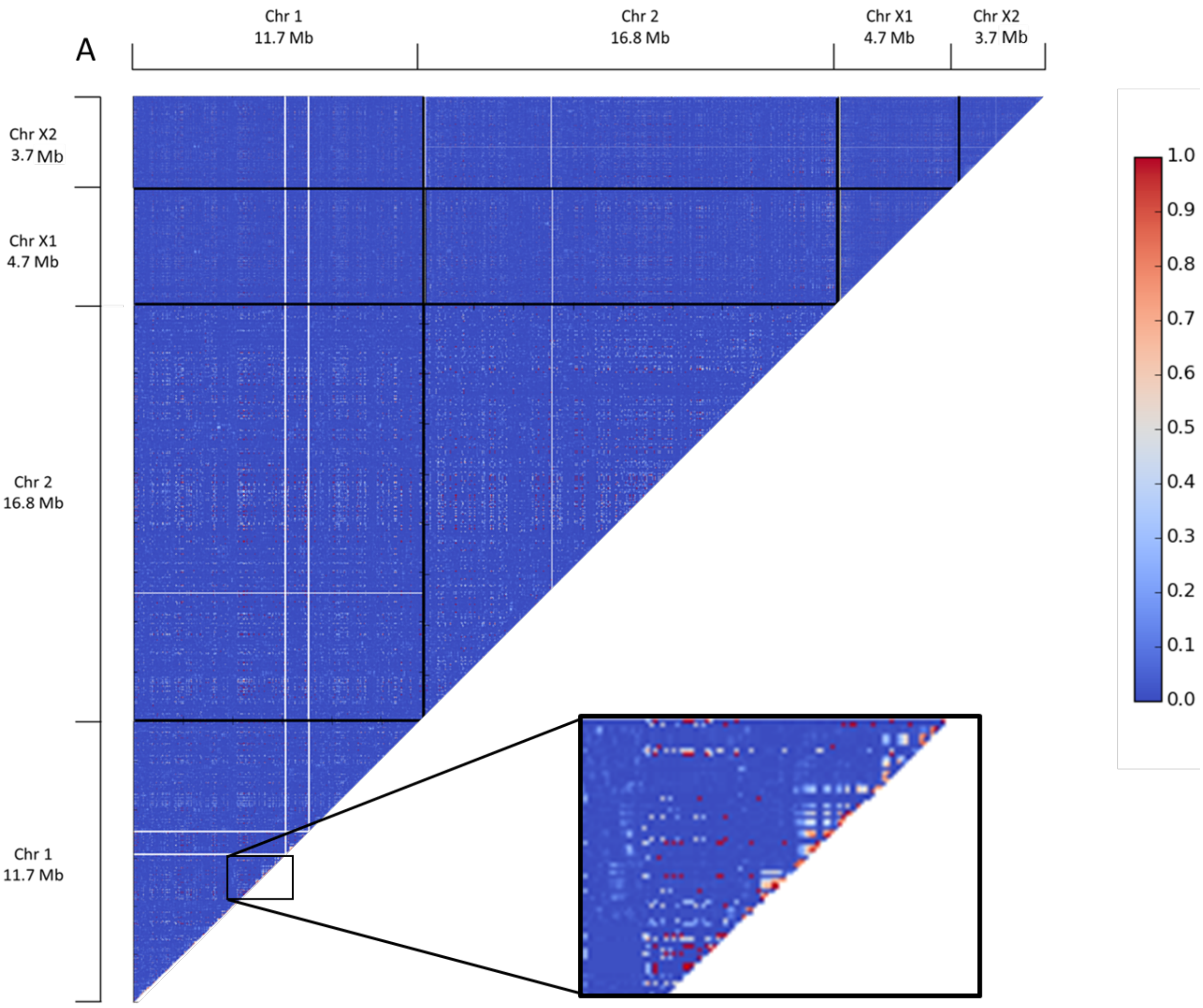

B

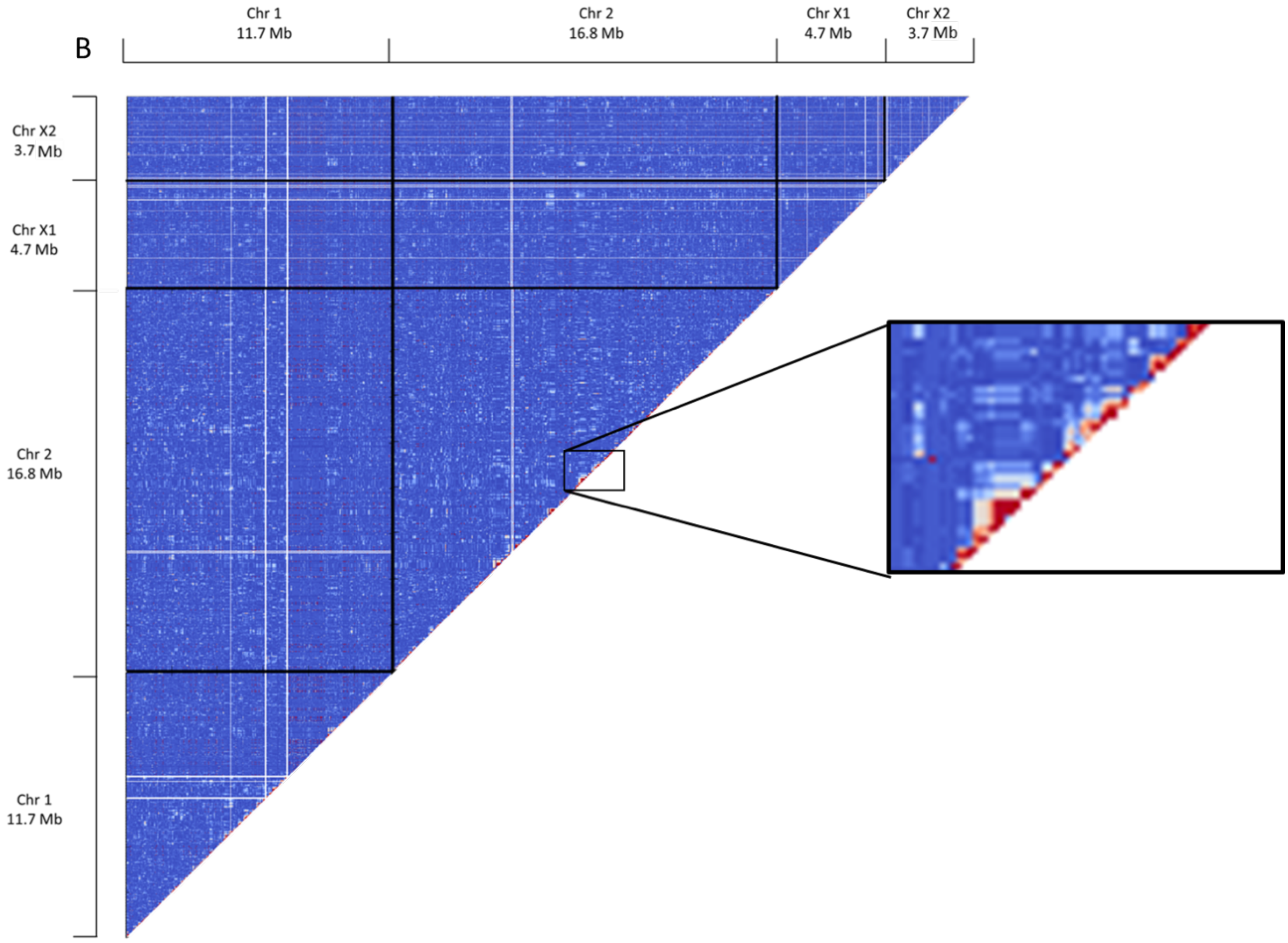

Chr 1
11.7 Mb
Chr 2
16.8 Mb
Chr X1
4.7 Mb
Chr X2
3.7 Mb
Chr X2
3.7 Mb
Chr X1
4.7 Mb
Chr 2
16.8 Mb
Chr 1
11.7 Mb

Supplementary Figure 10. Heatmaps of linkage disequilibrium in the *S. ratti* genome. Phasing of genotypes was carried out by Beagle (above the diagonal) or Shapeit (below the diagonal). X1 and X2 refer to the largest and second largest scaffolds of the X chromosome, respectively. Vertical and horizontal white lines in chromosome 1 represent two megabase long tracts of Ns that separate the three X chromosome scaffolds, whose genomic order is not known.

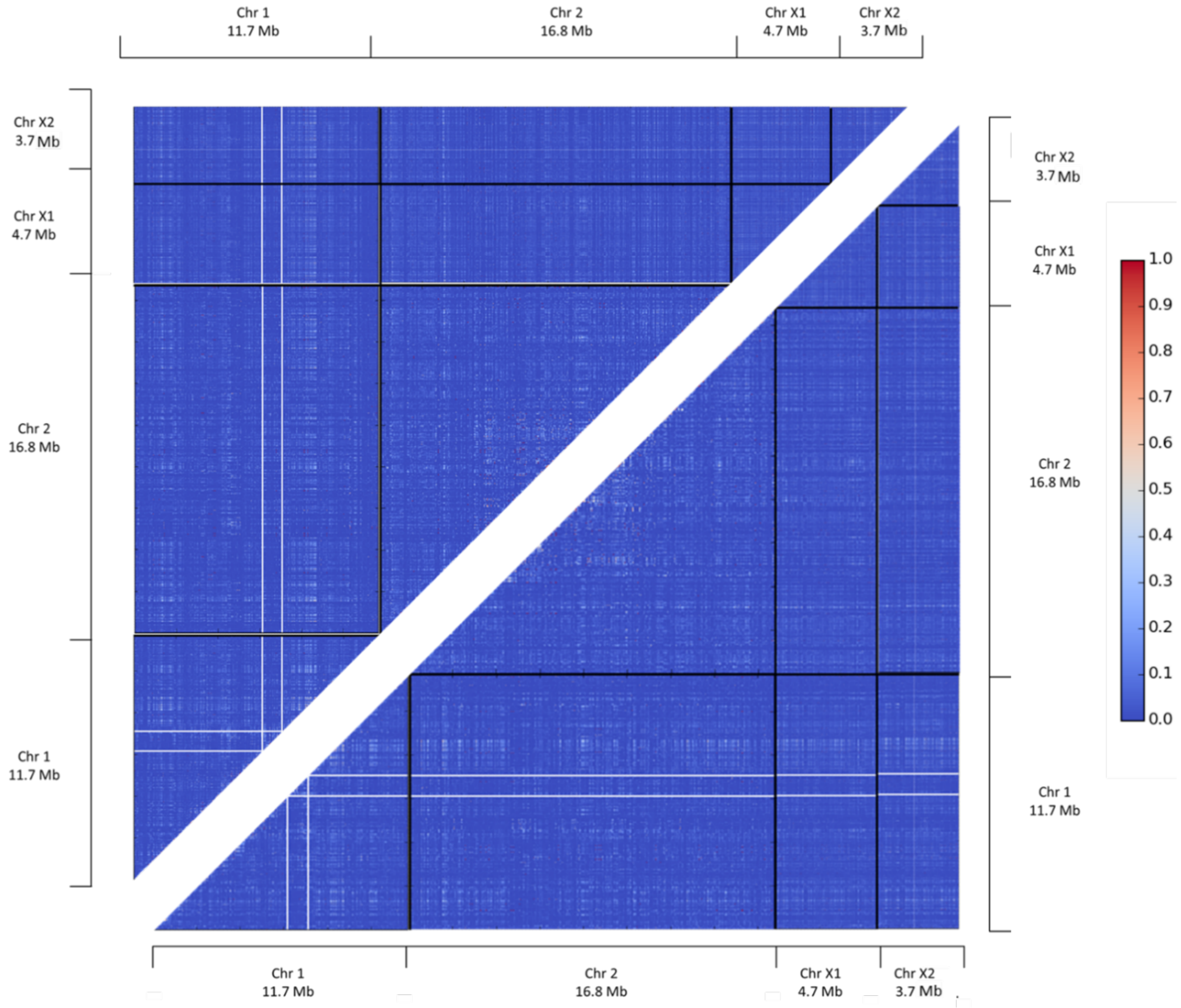

Supplementary Figure 11. Model of clade ancestry. There is a common ancestor of all parasites. From this ancestor there are then comparatively few generations of asexual reproduction, and so comparatively few mutations, leading to the ancestor of each clade. This is shown as Clade 1's common ancestor having a red mutation, Clade 3's common ancestor a blue mutation. Each clade than has, comparatively, many generations of asexual reproduction and many mutations are acquired. Considering LD, there will high LD among genotypes of Clade 1, and high LD among genotypes of Clade 3, but the LD of all Clade 1 and 3 genotypes together will be comparatively lower.

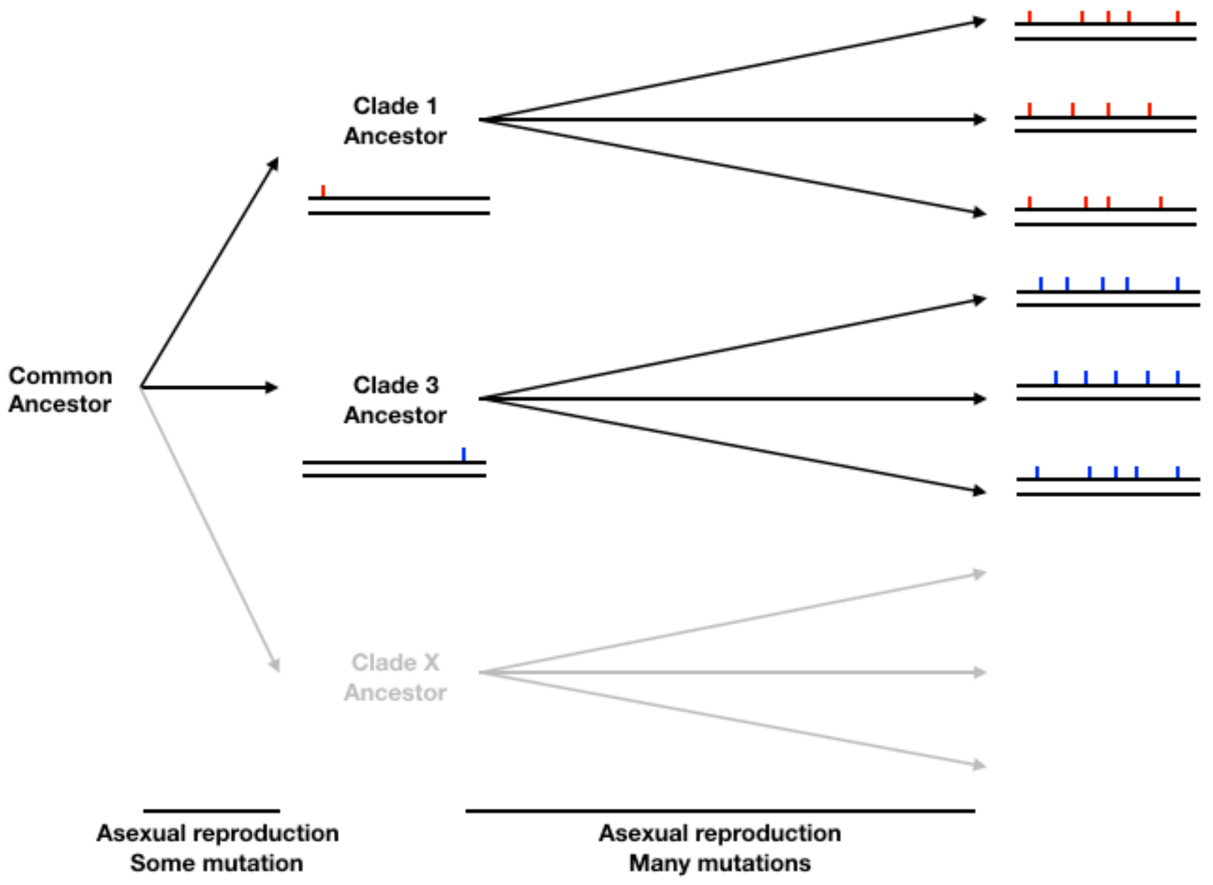

Supplementary Figure 12. PCA analysis of *S. ratti* parasites. Projections of principal components (PC) 1 and 2 of the 90 parasites from the three samples sites and the 10 isofemale lines, where PCs 1 and 2 explained 91% of the variance. Individuals are coloured according to (A) sampling site and (B) neighbour-joining dendrogram clades (**Figure 3**) with the colour coding scheme shown in each panel.

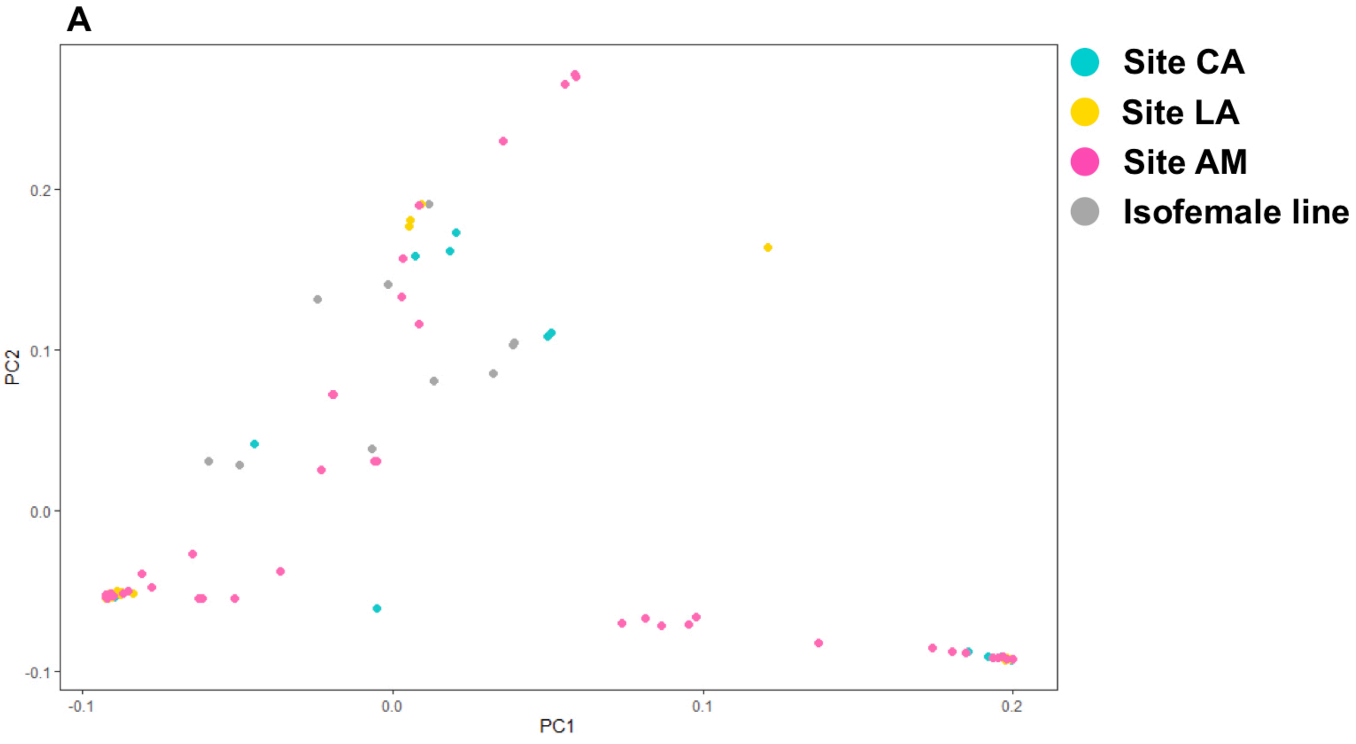

B

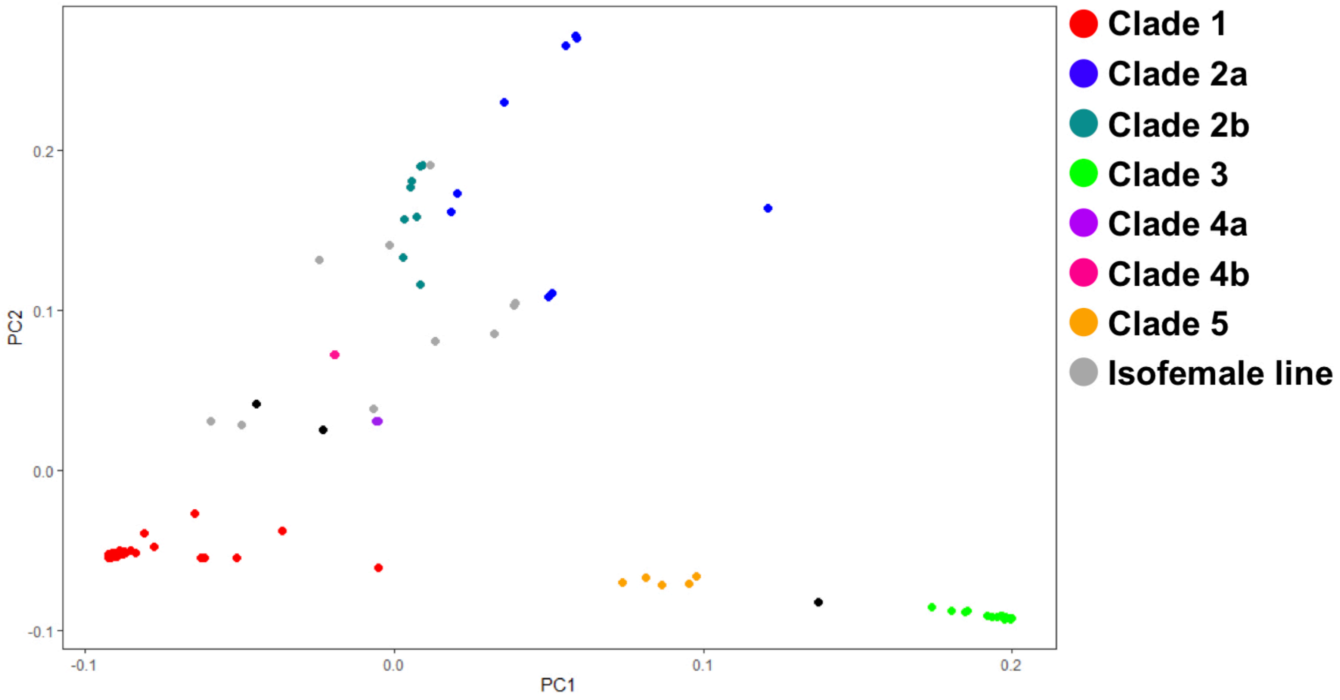

0.2
0.1
0.0
-0.1
PC2
-0.1
0.0
0.1
0.2
PC1
Clade 1
Clade 2a
Clade 2b
Clade 3
Clade 4a
Clade 4b
Clade 5
Isofemale line

Supplementary Figure 13. Minimum spanning *S. ratti* mitochondrial haplotype maps. Individual pellets are shown by their sampling site (AM, CA, LA) and the number preceding the underscore; the number after the underscore is the unique identifier of that worm. Haplotypes represented by multiple individuals are denoted by single letters. Four mitochondrial clades (A – D) are evident. Individual worms are coded either by the sampling site from which they were obtained or by the nuclear clade to which they belong (**Figure 3**). Mitochondrial clades A, B and C contained individuals from all 3 sampling sites, though at different rates. Mitochondrial clade A contains all individuals from nuclear clades 3 and 5 as well as one individual from nuclear clade 2b. Mitochondrial clade B contains individuals from nuclear clades 1, 2a and 4b. Mitochondrial clade C contains only individuals from nuclear clade 2b. The two individuals of nuclear clade 4a appear as intermediate between mitochondrial clades B and C in the haplotype map and so are designated as minor mitochondrial clade D.

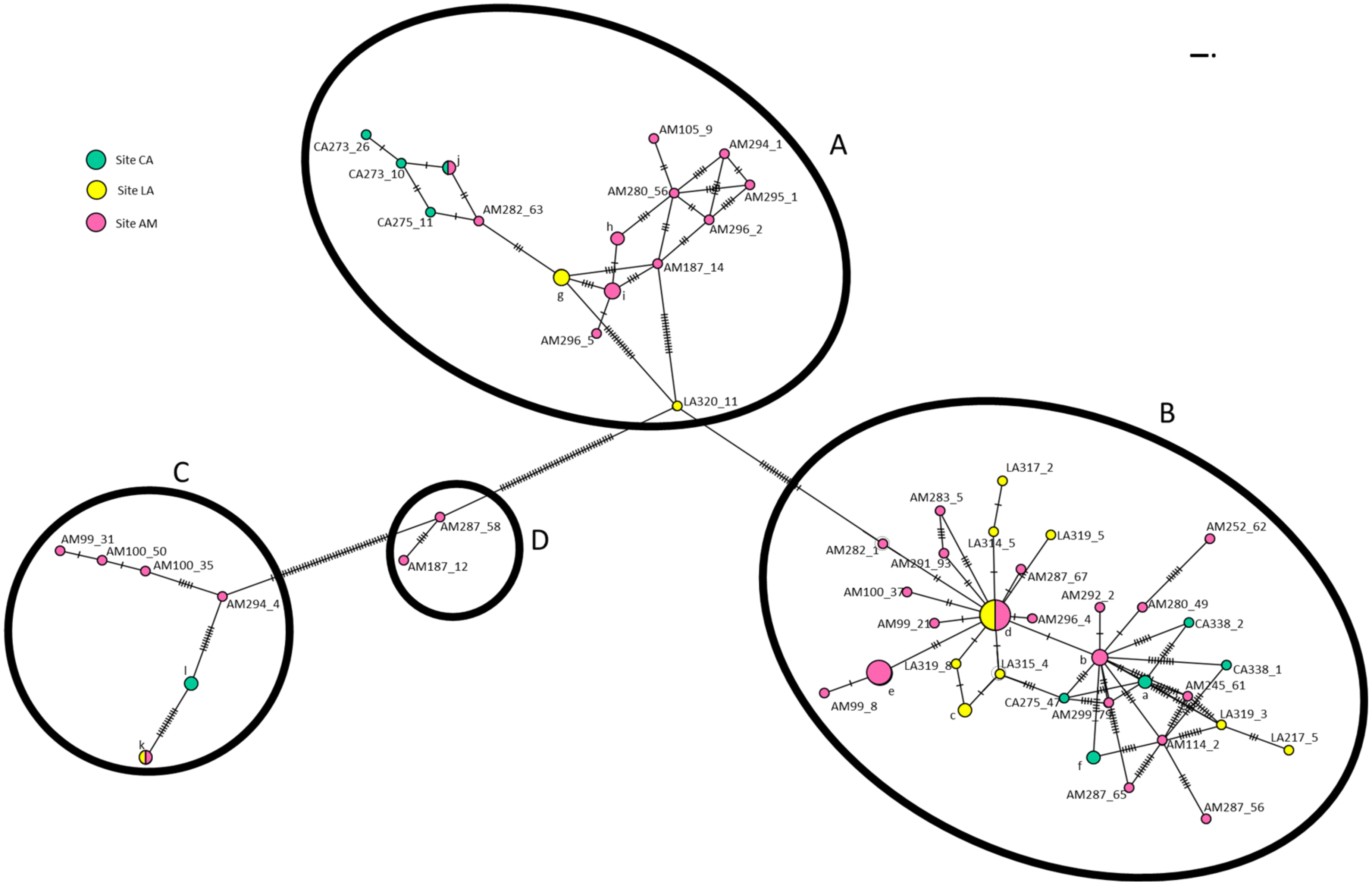
Site CA
Site LA
Site AM
A
B
C
D
CA273_26
CA273_10
CA275_11
AM282_63
AM105_9
AM294_1
AM280_56
AM295_1
AM296_2
AM187_14
AM296_5
LA320_11
AM99_31
AM100_50
AM100_35
AM294_4
AM287_58
AM187_12
LA317_2
AM283_5
LA319_5
AM252_62
AM282_1
AM291_93
AM287_67
AM100_37
AM292_2
AM280_49
AM296_4
AM99_21
CA338_2
LA319_8
LA315_4
CA338_1
AM245_61
CA275_47
AM299_79
LA319_3
LA217_5
AM114_2
AM99_8
AM287_65
AM287_56

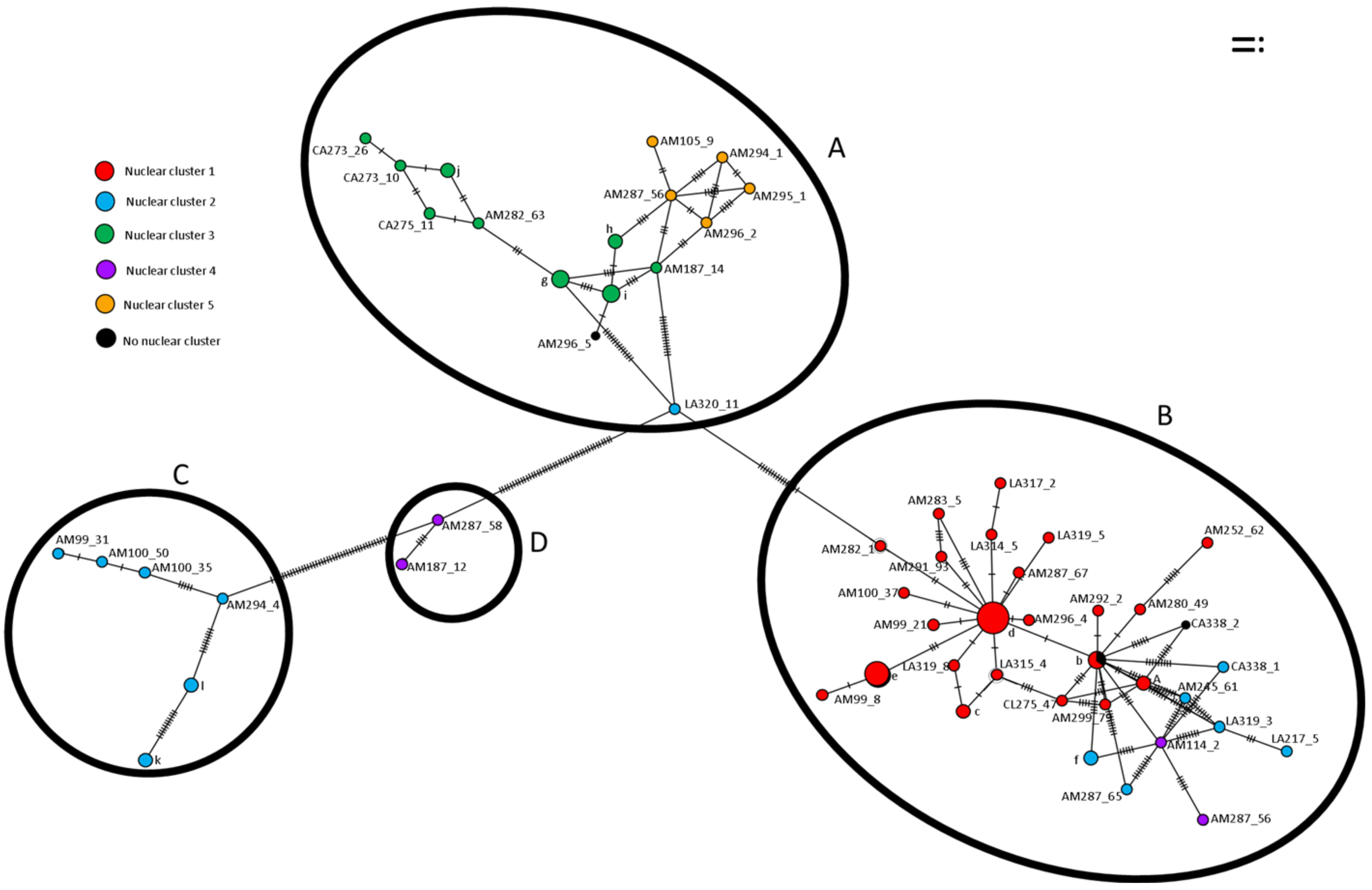

Nuclear cluster 1
Nuclear cluster 2
Nuclear cluster 3
Nuclear cluster 4
Nuclear cluster 5
No nuclear cluster
A
B
C
D
CA273_26
CA273_10
CA275_11
AM282_63
AM105_9
AM294_1
AM287_56
AM295_1
AM296_2
AM187_14
AM296_5
LA320_11
AM99_31
AM100_50
AM100_35
AM294_4
AM287_58
AM187_12
LA317_2
AM283_5
LA314_5
LA319_5
AM282_1
AM291_93
AM287_67
AM252_62
AM100_37
AM292_2
AM280_49
AM99_21
AM296_4
CA338_2
LA319_8
LA315_4
CA338_1
AM99_8
CL275_47
AM245_61
AM299_75
LA319_3
AM114_2
LA217_5
AM287_65
AM287_56

Supplementary Figure 14. Ritland and Lynch pairwise relatedness values of rats within sampling sites. The x-axis is in increments of 0.1, with only the upper limit shown. At each site there is a right-hand skew to these distributions; Shapiro-Wilkes test for normality statistics W = 0.89 for site AM, W = 0.9. for site CA and W = 0.87 for site LA, P > 0.0001 in all cases.

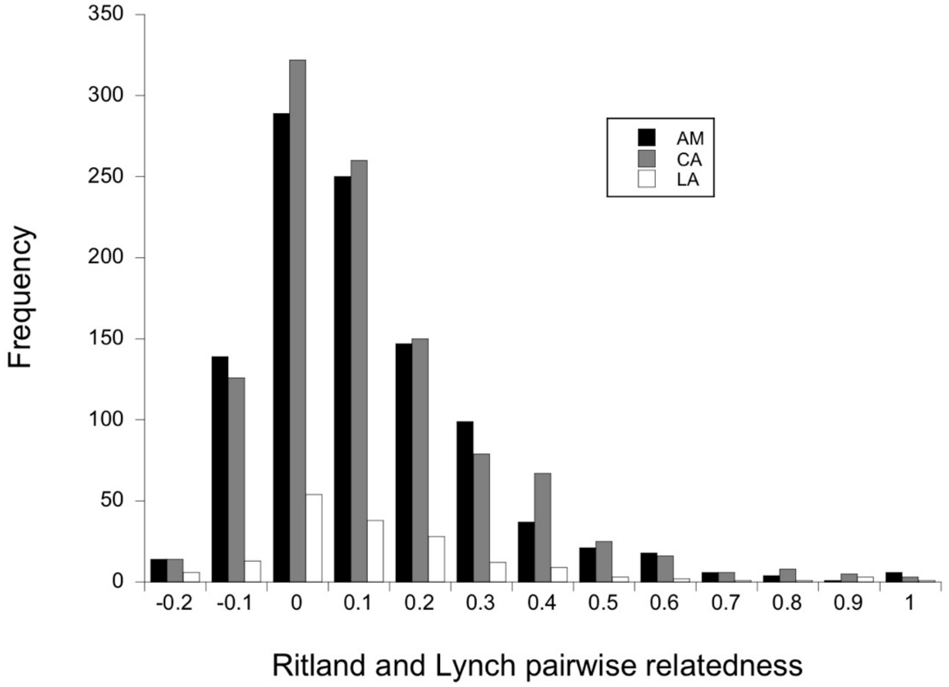

Supplementary Figure 15. ***S. ratti*** **neighbour-joining dendrograms of 10 isofemale lines and 90 larvae collected from the three sample sites.** Isofemale lines are prefixed by ED (**Supplementary Table 4**). Five of the isofemale lines (derived from parasites collected from the southern UK in 1989-90, and Japan in 1990) are within clade 2b, which is the most diverse of the clades, but the other 5 isofemale lines are in clades 1, 2a and 4.

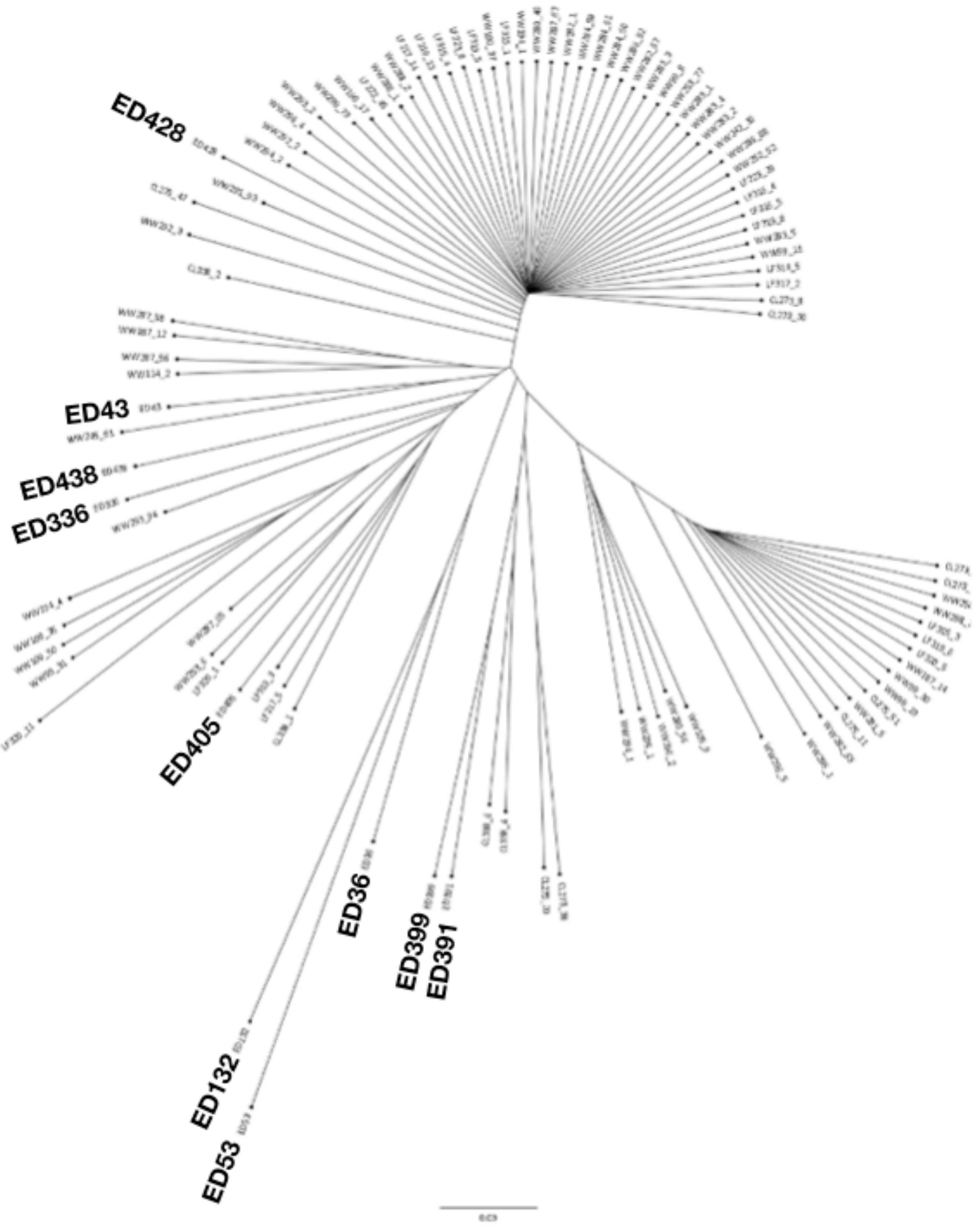

Supplementary Figure 16. The distribution of SNPs across the *S. ratti* genome. The distribution of SNPs for all 90 parasites for discrete, 10 kb windows, each represented by a vertical bar, for chromosomes 1, 2 and the two scaffolds of the X chromosome.

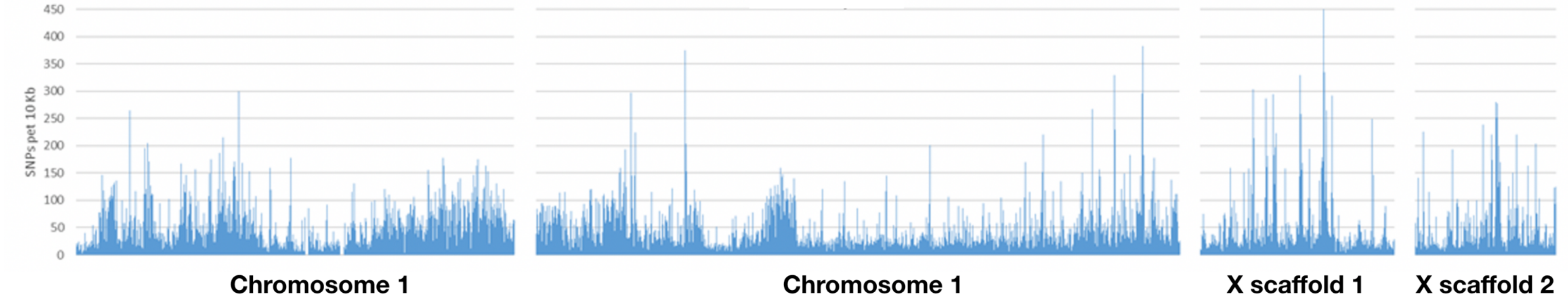

Supplementary Figure 17. Neighbour-joining dendrograms based on five expansion clusters. Clusters, 6, 7, 8, 12 and 14 are the clusters from which no genes were excluded due to concerns about genome assembly. Each tree was calculated based on the entire sequence from the start to the end of each cluster (excluding flanking regions). Clades (1 – 5) and sub-clades (2a and 2b, 4a and 4b) are defined based on the whole genome dendrogram (**Figure 3**). Individual parasites are marked with circles coloured according to their (sub-)clade in the whole genome dendrogram. Branch lengths are relative such that the distance between the two most distant individuals is 1, and so absolute distance differs among the panels.

Expansion Cluster 6

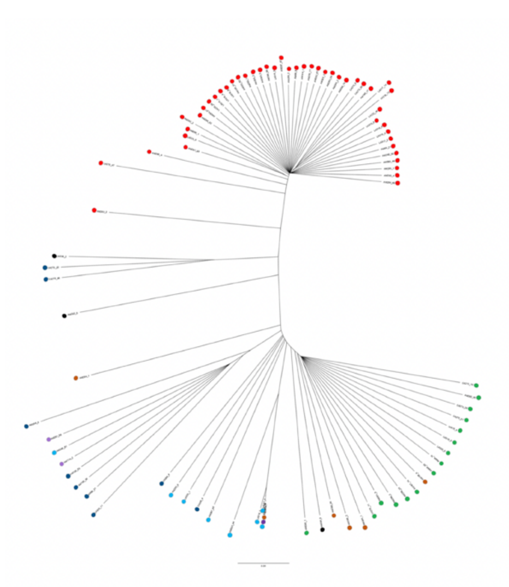

Expansion Cluster 7

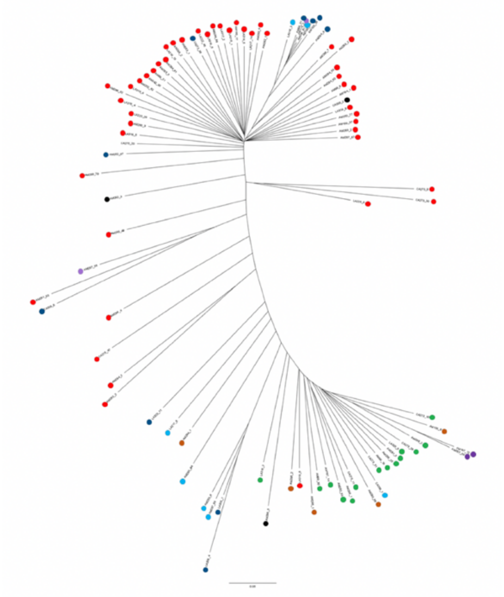

Expansion Cluster 8

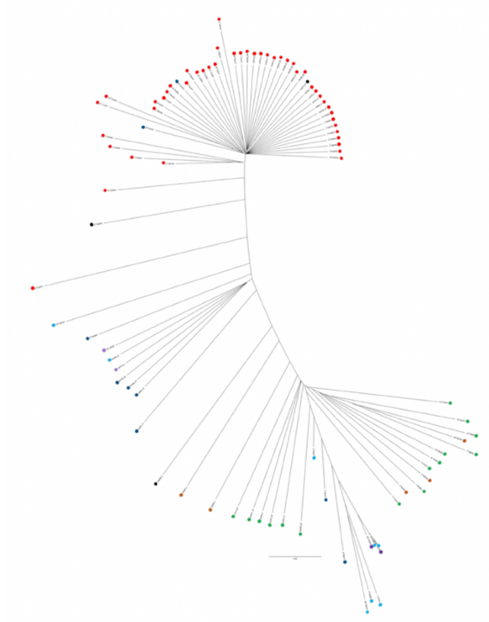

Expansion Cluster 12

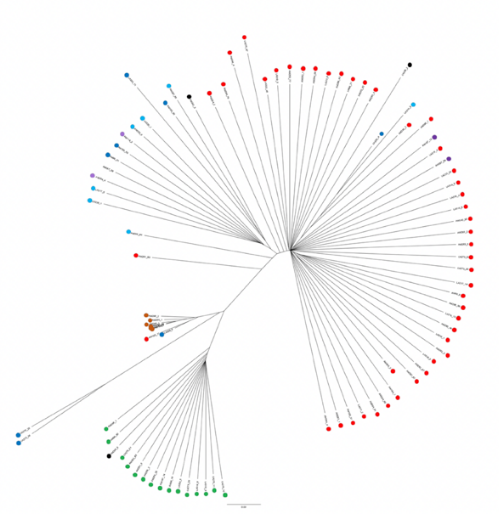

Expansion Cluster 14

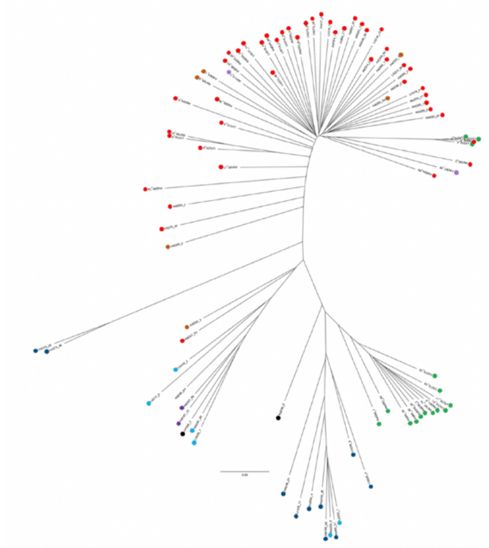

Supplementary Figure 18. Correlation of read depth and GC content for 90 *S. ratti* larvae. GC content is measured in non-overlapping 10 kb regions. Correlation ρ = 0.783, *df* = 4,008, $P < 0.00001$.

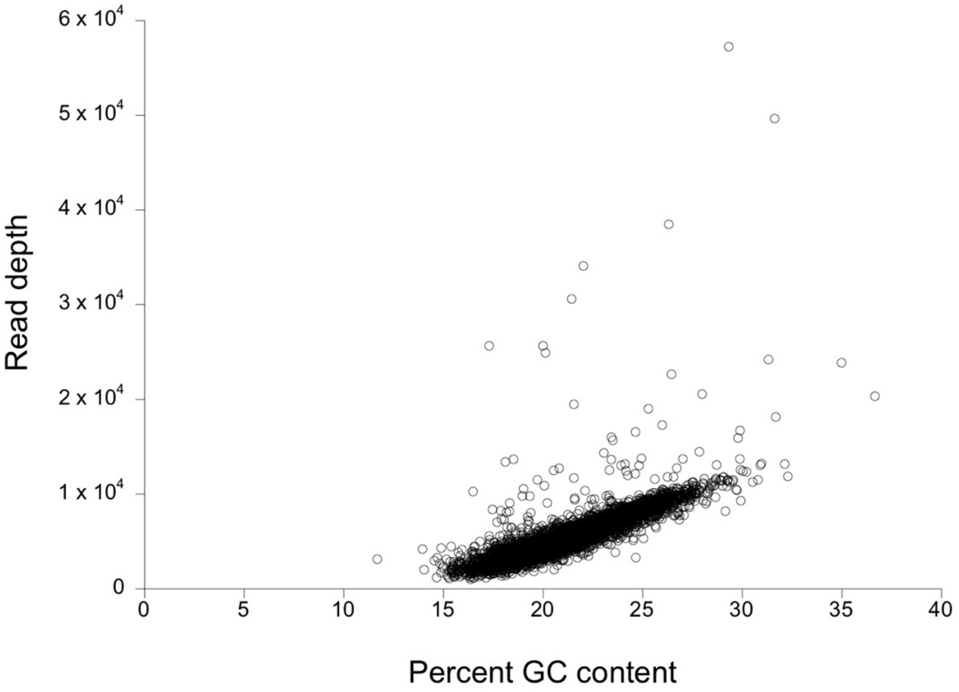

Supplement: Supplementary file 1 — Supplementary Information [file 41467_2023_42250_MOESM1_ESM.pdf]
