# Supplementary material for: The parasitic nematode Strongyloides ratti exists predominantly as populations of long-lived asexual lineages
Source: Nat Commun. 2023 Oct 13;14:6427. doi: 10.1038/s41467-023-42250-1 (PMC10575991; doi:10.1038/s41467-023-42250-1)
Supplement: Supplementary file 8 — Description of Additional Supplementary Files [file 41467_2023_42250_MOESM8_ESM.pdf]

Title: Supplementary Data 1. Genes in highly variable 10 kb regions.
Description: Region lists the 61 regions (region 1 does not contain any genes); Gene gives the gene's designation; Predicted function is the WormBase ParaSite description of each gene, where Astacin-like metalloendopeptidase is abbreviated to Astacin; Coding SNPs per kb is the number of SNPs per kb within the coding sequence of that gene; SNP type is the absolute number of synonymous (S), nonsynonymous (NS) and STOP codon-causing SNPs; Expression is taken from Hunt *et a!.*, 2016, which compared the expression of genes between the parasitic female and free-living female morph, and where the expression is one log_2 fold more in the parasitic female morph (Parasitic), free-living female morph (Free-living), not different (Same), or not listed (Unlisted); Expansion cluster is expansion cluster or associated flanking region a gene belongs to, if any. Some genes are marked as "discarded" because they had poor underlying assembly according to Gap5 analysis of expansion clusters and flanking regions and so were discounted from further analysis.

Title: Supplementary Data 2. The hundred most parasitic genes.
Description: Gene gives the gene's designation; Predicted function is the WormBase ParaSite description of each gene, where Astacin-like metalloendopeptidase is abbreviated to Astacin; Coding SNPs per kb is the number of SNPs per kb within the coding sequence of that gene; SNP type is the absolute number of synonymous (S), nonsynonymous (NS) and STOP codon-causing SNPs; Fold change is the log_2 difference in expression of the gene between the parasitic female and free-living female morphs taken from Hunt *et a!.,* 2016, here shown as positive values indicating greater expression in the parasitic female morph; Expansion cluster is expansion cluster or associated flanking region a gene belongs to, if any; Variable region is as defined in **Supplementary Data 1**. Some genes are marked as "discarded", because they had poor underlying assembly according to Gap5 analysis of expansion clusters and flanking regions and so were discounted from analyses.

Title: Supplementary Data 3. The hundred most free-living genes.
Description: Gene gives the gene's designation; Predicted function is the WormBase ParaSite description of each gene; Coding SNPs per kb is the number of SNPs per kb within the coding sequence of that gene; SNP type is the absolute number of synonymous (S), nonsynonymous (NS) and STOP codon-causing SNPs; Fold change is the log_2 difference in expression of the gene between the parasitic female and free-living female morphs taken from Hunt *et a!.,* 2016, here shown as positive values indicating greater expression in the free-living female morph; Expansion cluster is expansion cluster or associated flanking region gene belongs to, if any; Variable region is as defined in **Supplementary Data 1**.

Title: Supplementary Data 4. *S. ratti* expansion clusters, revised after further inspection of genome assembly in the cluster region.
Description: Region shows the numbered Flanking Regions (FR) and Expansion Clusters (EC); Gene the genes present within these regions; Predicted function is the WormBase ParaSite's description of the gene; Coding SNPs per kb is the number of SNPs per kb within the coding sequence of that gene; SNP type is synonymous (S), non-synonymous (NS) or STOP-causing (STOP); Expression shows whether the gene is upregulated (with a difference in expression of log_2-fold of at least 1 being considered upregulation) in the parasitic adult female morph (Parasitic), the free-living adult female morph (Free-living), or not differentially expressed (Same), all taken from Hunt *et a!.,* 2016, or unlisted there.

Title: Supplementary Data 5. Accessions of the 90 individual larvae in the European Nucleotide Archive (ENA) (https://www.ebi.ac.uk/ena/browser/home).

Description: Worm Identifier is the name of each larva referred to in this paper, and its respective ENA Sample Accession reference.

References

Hunt V.L., Asai I.J., Coghlan A., Reid A.J., Holroyd N., Foth B.J., *et al.* (2016). The genomic basis of parasitism in the *Strongyloides* clade of nematodes. *Nature Genetics* **48:**299-307
